# Comprehensive Analysis Reveals Dynamic and Evolutionary Plasticity of Rab GTPases and Membrane Traffic in *Tetrahymena thermophila*

**DOI:** 10.1371/journal.pgen.1001155

**Published:** 2010-10-14

**Authors:** Lydia J. Bright, Nichole Kambesis, Scott Brent Nelson, Byeongmoon Jeong, Aaron P. Turkewitz

**Affiliations:** 1 Department of Molecular Genetics and Cell Biology, The University of Chicago, Chicago, Illinois, United States of America; 2 Department of Chemistry and Nano Science, Ewha Womans University, Seoul, Korea; Washington University School of Medicine, United States of America

## Abstract

Cellular sophistication is not exclusive to multicellular organisms, and unicellular eukaryotes can resemble differentiated animal cells in their complex network of membrane-bound structures. These comparisons can be illuminated by genome-wide surveys of key gene families. We report a systematic analysis of Rabs in a complex unicellular Ciliate, including gene prediction and phylogenetic clustering, expression profiling based on public data, and Green Fluorescent Protein (GFP) tagging. Rabs are monomeric GTPases that regulate membrane traffic. Because Rabs act as compartment-specific determinants, the number of Rabs in an organism reflects intracellular complexity. The *Tetrahymena* Rab family is similar in size to that in humans and includes both expansions in conserved Rab clades as well as many divergent Rabs. Importantly, more than 90% of Rabs are expressed concurrently in growing cells, while only a small subset appears specialized for other conditions. By localizing most Rabs in living cells, we could assign the majority to specific compartments. These results validated most phylogenetic assignments, but also indicated that some sequence-conserved Rabs were co-opted for novel functions. Our survey uncovered a rare example of a nuclear Rab and substantiated the existence of a previously unrecognized core Rab clade in eukaryotes. Strikingly, several functionally conserved pathways or structures were found to be associated entirely with divergent Rabs. These pathways may have permitted rapid evolution of the associated Rabs or may have arisen independently in diverse lineages and then converged. Thus, characterizing entire gene families can provide insight into the evolutionary flexibility of fundamental cellular pathways.

## Author Summary

Single-celled organisms appear simple compared to multicellular organisms, but this may not be true at the level of the individual cell. In fact, microscopic observations suggest that protists can possess networks of organelles just as elaborate as those in animal cells. Consistent with this idea, recent analysis has identified large families of genes in protists that are predicted to act as determinants for complex membrane networks. To test these predictions and to probe relationships between cellular structures across a wide swath of evolution, we focused on one gene family in the single-celled organism *Tetrahymena*. These genes control the traffic between organelles, with each gene controlling a single step in this traffic. We asked three questions about each of 56 genes in the family. First, what is the gene related to in humans? Second, under what conditions is the gene being used in *Tetrahymena*? Third, what is the role of each gene? The results provide insights into both the dynamics and evolution of membrane traffic, including the finding that some pathways appearing both structurally and functionally similar in protists and animals are likely to have arisen independently in the two lineages.

## Introduction

Cells can respond to and shape their environments by taking up and releasing macromolecules. Both in- and outbound transport is facilitated by a network of membrane-bound compartments that represent one of the hallmarks of eukaryotic cells [1]. Traffic through the network is highly regulated, and a convergence of data from structural, functional, and evolutionary studies demonstrate that gene families encode proteins functioning as conserved specificity determinants for endocytic and exocytic compartments [2], [3]. They do so primarily by controlling the formation, targeting and fusion of vesicles that transport cargo between compartments [4]. One family of key determinants are monomeric GTPases called Rabs, which function as molecular switches by interacting with membrane bilayers and diverse protein effectors in cycles controlled by GTP binding and hydrolysis [5].

A fundamental aspect of Rab function is that multiple Rabs, encoded as a gene family, are co-expressed within a single cell, and the individual family members are each targeted to a small subset of membrane compartments, where they interact with unique effectors [6]. In this manner, a cohort of Rabs can coordinately regulate a pathway consisting of sequential and distinct trafficking events [7]. It is therefore likely that gene duplications within the Rab family, followed by diversification into functionally-distinct variants, were key steps in the evolution of eukaryotic membrane complexity [8], [9]. This model is strongly supported by the tendency of Rabs associated with specific organelles, across a wide range of eukaryotes, to be most closely related to one another. For example, the Rab1 clade has remained highly conserved at the sequence level among all eukaryotic kingdoms; the corresponding proteins, where they have been characterized, are all associated with traffic between the endoplasmic reticulum and cis-Golgi [10]. These experimental findings coupled with phylogenetic parsimony reveal that Rab1 was already a determinant for this step in an early eukaryotic ancestor. It follows that the analysis of lineage-specific expansions or losses in conserved Rab clades can provide insights into the range of evolutionary paths that have led to modern cells.

Because of their central importance, Rabs have been studied in a variety of model and non-model organisms. As expected, the number of Rabs in an organism is generally related to organismal complexity [11], [12] including tissue-specific expression in multicellular organisms [13]. However, a surprising result emerging from several recently-sequenced genomes is that some unicellular organisms express a cohort of Rabs whose number equals or exceeds the 63 Rabs in humans [14]. An extreme example is the parasite *Entamoeba histolytica*, whose genome encodes 91 Rabs [15]. The sheer number of Rabs hints at an interesting discordance between organismal simplicity and cellular complexity, the latter facilitated at least in part by extensive, lineage-restricted Rab expansions. However, the roles of most *Entamoeba* Rabs are unknown, and the large Rab number in *Entamoeba* could endow the cell with flexibility, rather than structural complexity, if different subsets of the *Entamoeba* Rabs are expressed under the widely disparate conditions that are cyclically encountered by a parasite.

Ciliates have classically been recognized as unicellular organisms of great structural complexity including a wealth of membrane-bound compartments [16]–[22]. Recent sequencing of two ciliate genomes revealed that these organisms are similarly gene-rich [23], [24]. The free-living ciliate *Tetrahymena thermophila* has more than 20,000 genes and preliminary analysis suggested that these included 70 Rabs, raising the same issues discussed above for *Entamoeba* [23]. To shed light on the nature and evolution of unicellular cell complexity, we have taken a whole genome approach by considering the entire set of Rabs in *Tetrahymena*, including phylogenetic analysis, expression profiling, and localization of GFP-tagged variants in living cells. While many of the cellular pathways illuminated by this study represent ancient, highly conserved functions, the unbiased nature of our survey also uncovered a surprising level of both evolutionary and cell-stage flexibility within Rab GTPases, key determinants of membrane traffic.

## Results

### *Tetrahymena* encodes a set of predicted Rabs as extensive as those in many multicellular organisms

Beginning with amino acid sequences of human Rabs, we identified 56 Rabs in the ciliate *Tetrahymena thermophila*. This does not include seventeen putative Rabs that were reported in a previous survey [23], because these were found to lack the specific C-terminal residues to which prenyl residues are attached in authentic Rabs. These 17 predicted genes should therefore be reassigned as Rab-like proteins [25]. We then analyzed all putative *Tetrahymena* Rabs, including 3 that were not detected in the previous survey, for the presence of five Rab-specific motifs: IGVDF, KLQIW, RFRSIT, YYRGA and LVYDIT [26]. While many of the *Tetrahymena* Rabs have diverged to various degrees at some of these consensus sites, these genes are nonetheless clearly more similar to Rabs than to any other class of small GTPase, i.e., Ras, Rho/Rac, Sar/Arf or Ran (Figure S1; GenBank ID numbers provided in Table S1). Consistent with this, BLAST searches based on any of the 56 Rabs identified only other Rabs as best hits.

*Tetrahymena*, although a single-celled organism, therefore has a somewhat larger set of Rabs than in *D. melanogaster* or *C. elegans*, and a much larger set than is present in model fungi (*S. cerevisiae* or *S. pombe*) or parasitic protists such as *T. brucei* or *P. falciparum*, the latter belonging to the clade most closely related to Ciliates, the Apicomplexans(Table 1). The number of Rabs is similar, however, to those reported for some other single-celled organisms, such as *T. vaginalis*. The large number of predicted Rabs suggests that some protists maintain networks of membrane compartments that are at least similar in complexity to those in multicellular organisms. However, no comprehensive analysis of the Rabs in any of these organisms has been reported, and the large number of Rabs could reflect alternative expression of paralogs to optimize a relatively simple network of compartments for changes in environmental conditions or lifestages.

**Table 1:** The genomes of some unicellular organisms encode large numbers of predicted Rabs, similar to or exceeding those of multicellular organisms.

Organism	Predicted Rabs
*Homo sapiens*	63
*Arabidopsis thaliana*	57
*Drosophila melanogaster*	33
*Caenorhabditis elegans*	29
*Saccharomyces cerevisiae*	12
*Schizosaccharomyces pombe*	8
*Trypanosoma brucei*	16
*Plasmodium falciparum*	11
*Dictyostelium discoideum*	54
*Entamoeba histolytica*	91
*Trichomonas vaginalis*	65
*Tetrahymena thermophila*	56

Data are from: for *H. sapiens* [13]; *A. thaliana*, *C. elegans*, *S. cerevisiae* [11]; for *D. melanogaster* [13]; for *T. brucei* [81]; for *P. falciparum* [82]; for *D. discoideum*, *T. vaginalis* [14]; for *E. histolytica* [15]; for *T. thermophila* (this manuscript).

### Concurrent expression of most *Tetrahymena* Rabs indicates a large number of concurrent pathways

The transcript abundance of all *Tetrahymena* genes has been measured via whole genome microarrays, using mRNA samples derived from growing (medium and high density) and stationary cultures in growth medium, 7 successive time points during starvation, and 10 successive time points during conjugation [27]. This dataset is particularly useful since transcription plays a central role in the control of differential gene expression in *Tetrahymena* [28]. Using the publicly accessible database at http://tged.ihb.ac.cn, we found that the transcript abundance of all 56 Rabs is above background and, strikingly, 86% are in the highest-expressing class of genes in this organism [27]. Therefore, none is likely to be a pseudogene. Importantly, 94% of the Rabs are transcribed in growing cell cultures, indicating that membrane traffic in growing *Tetrahymena* involves more than 50 distinct Rab proteins. Thus the large number of Rab genes is likely to reflect a large number of pathways of membrane traffic that function concurrently in a complex cell, rather than a small number of pathways that are controlled by alternative Rab isoforms in a stage-specific fashion. This analysis also suggested that the majority of *Tetrahymena* Rabs could be meaningfully localized in cells from growing cultures.

Both morphological as well as some molecular studies have revealed extensive changes in membrane traffic when *Tetrahymena* are starved [29]–[32], and additional changes during conjugation including the elaboration of a cell-cell fusion zone and the creation of some, and loss of other, nuclei [33]–[35]. The expression data suggest that these changes involve two different phenomena with regard to Rab expression (Table S1). First, RabsD16 and D12 are not expressed in growing cultures but are greatly induced at specific stages in starvation or conjugation: the former at 6h of starvation, the latter most dramatically at 9h of starvation and at the beginning of conjugation (Figure S2). Secondly, many Rabs that are expressed in growing cells are redeployed in starvation or conjugation. In particular, many of the *Tetrahymena* Rabs that are expressed in growing cells are also expressed in starved or conjugating cells, and indeed most show even higher transcript levels in one of these conditions (65% in starved, and 27% in conjugating cells). However, because average translation efficiency is much greater in growing than in starved cells [36], the changes in transcript levels under different culture conditions do not necessarily reflect corresponding protein abundance. We therefore looked for peaks of Rab expression *within* each growth condition, as an indication that some Rabs might be determinants of precise stage-specific structures in starvation or conjugation. Strikingly, 20 Rabs show a discrete >2-fold expression peak at a distinct time point during starvation (Rabs D22, D16, D21, all near S6, i.e., the 6h starvation timepoint) or during conjugation (C0, i.e., the beginning of conjugation: Rabs D29, D7; C2, i.e, the 2h conjugation time point: Rabs 11A, D26, D33, D24, D7, D23, D31, D38, 21; C4: Rabs D35, 11C; C6: Rabs 4A, D13; C14: RabD34; C16: Rabs D21, D15; C18: Rabs D22, D16)(Table S1). The preponderance of relative Rab expression peaks at C2 corresponds with extensive membrane restructuring during pair formation. In addition, many Rabs show a large expression peak at S0; however, this time point is difficult to interpret because it corresponds to a change in the culture medium.

### Many Rabs can be assigned to known pathways of membrane traffic based on localization of GFP-fusions

**Figure 1:**
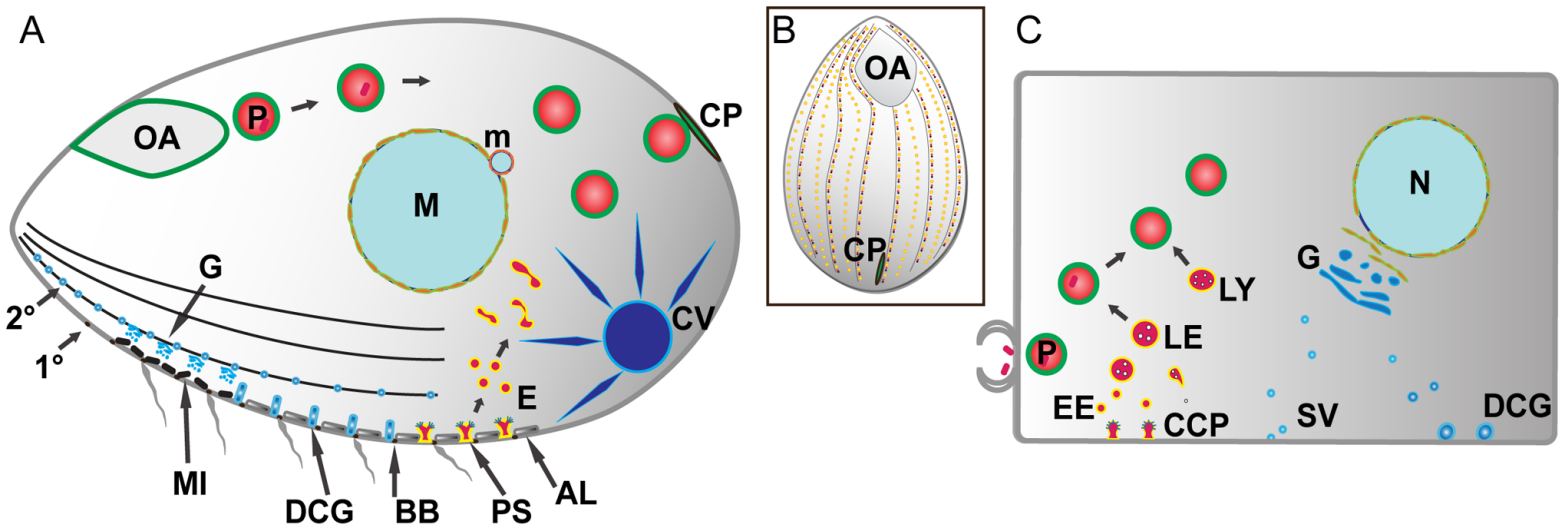
Pathways of membrane traffic in *Tetrahymena* and in animal cells. A. *Tetrahymena* cell cartoon. Cell length is ∼50µM; anterior to the left. A prominent pathway of phagocytosis begins at the anterior oral apparatus (OA), resulting in phagosomes (P) that eventually egest undigested material at the cytoproct (CP). The OA and CP exist as single-copy structures at fixed positions. Clathrin-mediated endocytosis occurs at multiple, regularly-spaced invaginations called parasomal sacs (PS), giving rise to endocytic vesicles (E). These endocytic vesicles coalesce in tubulovesicular endosomes in the cell posterior. Protein secretion occurs via the endoplasmic reticulum (not shown) and Golgi (G), which is present as single cisterna or short stacks near the cell periphery, close to mitochondria (MI). Some of the secretory cargo is packaged into dense core granules (DCG), which dock and subsequently undergo exocytosis at sites on 1° and 2° meridians (1° and 2°), which are cytoskeletal “ribs”. A prominent organelle, whose membrane dynamics are poorly understood, is the water-pumping contractile vacuole (CV). Other structures detailed in the *Tetrahymena* cell: AL: alveolae: a calcium-storage compartment. BB: ciliary basal bodies. M: macronucleus (polyploidy vegetative nucleus). m: micronucleus (diploid germline nucleus). B. All surface features in *Tetrahymena* except the OA and CP are repeated at fixed positions in a grid that can be defined by the array of 1° and 2° meridians. C. Analogous structures and pathways are diagrammed in a generalized mammalian cell. The abbreviations are the same as in the *Tetrahymena* cell with the additional structures: CCP: clathrin-coated pit. EE: early endosomes. LE: late endosomes. LY: lysosomes. SV: secretory vesicles.

Figure 1 gives an overview of known or inferred pathways of membrane traffic in *Tetrahymena*, as well as those in a generalized mammalian cell. To identify *Tetrahymena* Rabs dedicated to specific pathways or structures, we expressed them individually as GFP-tagged proteins and determined the localization in living *Tetrahymena*. GFP-Rab expression was induced at the lowest level permitting clear visualization, and confirmed using Western blotting of *Tetrahymena* lysates (Figure S3, with two exceptions for Rabs expressed at low levels). For practical reasons, most Rabs were localized solely in growing cultures. In preliminary studies, we found evidence that Rabs whose endogenous expression was largely limited to starvation or conjugation were de-localized when expressed in growing cells (Figure S2), and therefore we did not include that small set of Rabs in this study. We captured time-lapse and/or full z-stacks of cells expressing each of the Rabs in living, immobilized cells. Movies of all Rabs described in this paper are publicly accessible at tetrahymenacell.uchicago.edu. A number of the Rabs were seen to associate with more than one structure (Table S2). In these cases we considered the stronger signal as primary, and the data below are organized based on these primary signals. In rare cases, we saw equally strong labeling of two structures.

### An active pathway of phagocytosis involves a large subset of the *Tetrahymena* Rabs

**Figure 2:**
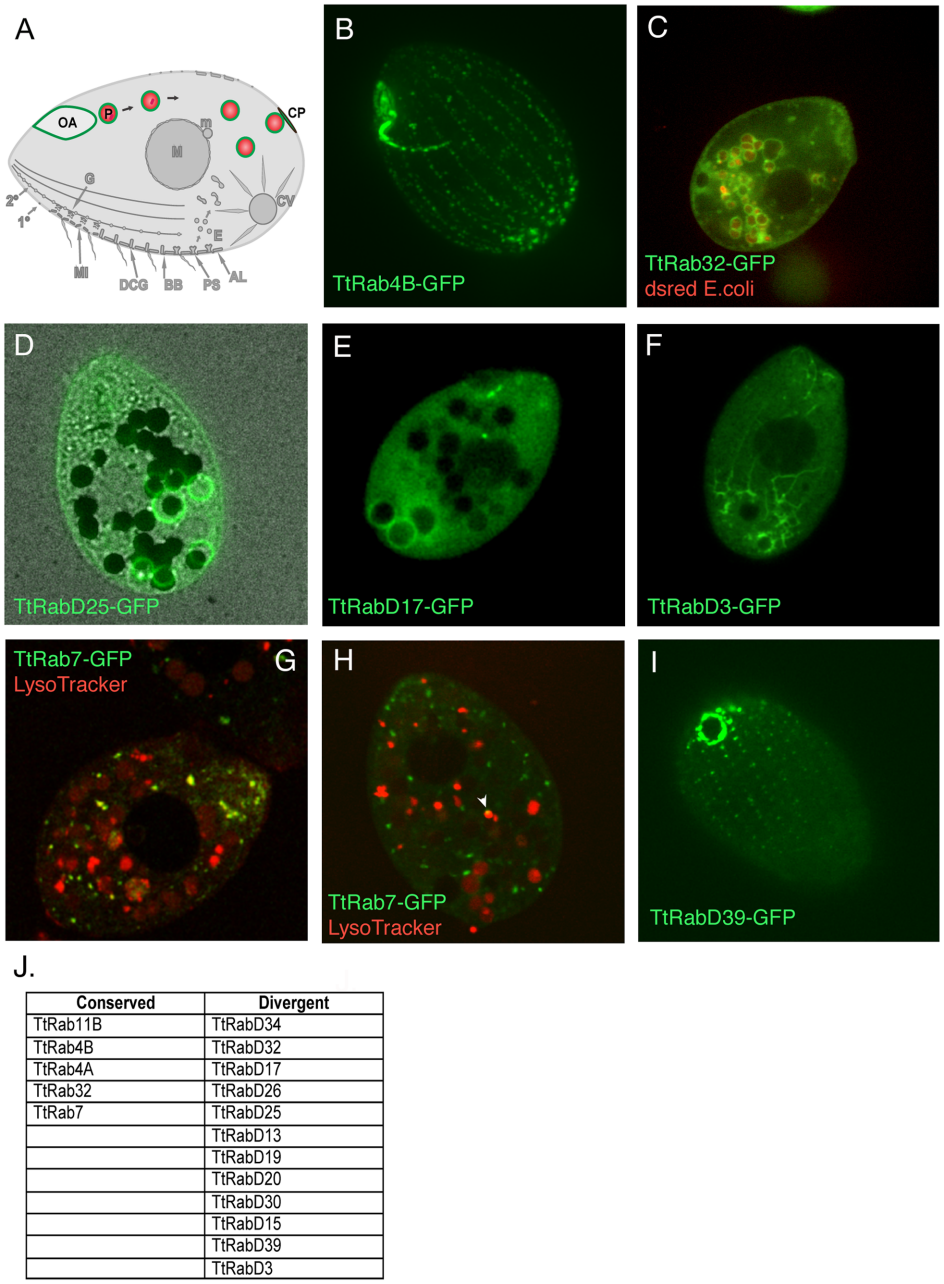
Rabs associated with phagocytosis. A. Phagosomes (P) form at the oral apparatus (OA), undergo maturation and transport in the cytoplasm in a process involving membrane fusion and fission, and eventually fuse with the plasma membrane at the cytoproct (CP). This is followed by membrane retrieval and transport of endosomes back to the oral apparatus. B–I. All panels are confocal images of live cells following induction of GFP-Rab expression for 2 hours in S media, unless otherwise indicated. GFP-TtRabs are associated with distinct structures: (B) oral apparatus; (C) all phagosomes; (D) selected phagosomes; (E) cytoproct-associated phagosomes; (F) cytoplasmic microtubules extending from posterior phagosomes to the cell anterior. Phagosomes in C–F contain either dsRed-expressing *E. coli* (red) or india ink particles (black). G. For cells in SPP medium, LysoTracker (red) labels both phagosomes (larger red vacuoles) and smaller vesicles (red and yellow) that are likely to be lysosomes. TtRab7 colocalizes with the latter, which are concentrated in the anterior cytoplasm. H. In cells cultured in S medium, the colocalization of GFP-TtRab7 with LysoTracker is limited chiefly to very small puncta at the periphery of phagosomes (arrowhead). I. cytoproct. The *Tetrahymena* shown are ∼50×20µM. J. The full set of TtRabs associated with phagocytosis.

Ciliates like *Tetrahymena* can feed via the phagocytic uptake of bacteria or other particulate matter via a cavity at the cell anterior called the oral apparatus (Figure 2A) [37], [38]. Bacteria are swept into the oral apparatus by cilia at the oral apparatus rim, and ingested at the base. Following ingestion, the bacteria are digested via phagosome maturation resembling that in mammalian cells, including fusion of phagosomes with both early and late endocytic compartments. The final stage of phagosome maturation, which has no precise equivalent in mammalian cells, entails docking and fusion with a structure at the cell cortex called the cytoproct, which results in the egestion of any residual material in the phagosome lumen. Egestion is followed by a burst of membrane retrieval, in a process (documented by EM in another ciliate, *Paramecium micronucleatum*) that results in the rapid appearance of endosomes in the cytoproct region. These endosomes appear to be transported anteriorly along cytoplasmic microtubules, where they may contribute membrane during the formation of phagocytic vesicles from the oral apparatus [39].

Remarkably, almost 1/3^rd^ of the Tetrahymena Rabs were found to be associated with some aspect of the phagocytic pathway (examples shown in Figure 2; full set in Figure S4). Five of these are localized primarily to structures at or near the oral apparatus, while eleven are localized to phagosomes that can be unambiguously identified in cells that have ingested either fluorescent bacteria or India ink (Figure S4) (Table 2). Some GFP-Rab fusion proteins are associated with the entire cohort of phagosomes in a cell (Figure 2C), but many of the Rabs are associated with only a subset of the phagosomes (Figure 2D), which may correspond to stages in maturation. Consistent with the specialization of the cytoproct as a zone of membrane fusion and retrieval, three of the phagosome Rabs are restricted to phagosomes that are localized at or near the cytoproct (Figure 2E), and time-lapse movies of cells expressing these GFP-Rab fusions allowed us to directly visualize phagosome egestion and retrieval (Videos S1, S2, S3, S4).

**Table 2:** Summary of all TtRabs based on primary localization pattern.

	Rab
**Parasomal sacs**	TtRabD5
**Endocytic vesicles**	**TtRab21**
	**TtRab22A**
	TtRabD4
	TtRabD28
**Posterior (recycling?) endosomes**	**TtRab11A**
	TtRabD35
	TtRabD27
	TtRabD24
**Lysosomes/phagosomes**	**TtRab7**
**Oral apparatus**	**TtRab11B**
	**TtRab4B**
	TtRabD34
	TtRabD32
	TtRabD17
**All phagosomes**	**TtRab32**
**Selected phagosomes**	TtRabD26
	TtRabD25
	TtRabD13
	TtRabD17
	TtRabD3
	TtRabD15
**Cytoproct-localized phagosomes**	TtRabD19
	TtRabD20
	TtRabD30
**Cytoproct region**	**TtRab4A**
	TtRabD39
**Contractile vacuole Rabs**	TtRabD2
	TtRabD10
	TtRabD14
**ER-to-Golgi**	**TtRab1**
	TtRabD33
**Golgi**	**TtRab6C**
	**TtRab6D**
	**TtRab6B**
	**TtRab6A**
	TtRabD38
**Dense core granule docking**	TtRabD41
**Basal bodies**	TtRabD23
**Cortical cytoskeleton**	TtRabD36
	TtRabD40
	**TtRab31**
**Plasma membrane vicinity**	TtRabD18
	TtRabD29
**Nuclear envelope**	TtRabD31
**Indeterminate structures**	TtRabD6
	TtRabD7
	TtRabD9
	TtRabD11
	TtRabD21
	TtRabD12
	TtRabD16
	TtRabD1
**Not cloned**	**TtRab11C**
	TtRabD8
	TtRabD22
	TtRabD37

TtRab7, the Rab7 homolog, was previously associated with phagosomes in a proteomics study [37]. We found that this protein localizes both to lysosomes (as defined by co-labeling with LysoTracker) and to small puncta on the surface of phagosomes (Figure 2G and 2H).

TtRabD17 localizes to phagosomes in the vicinity of the cytoproct but also localizes strongly to the oral apparatus, suggesting the possibility of long-distance transport between these structures. Consistent with this, the live imaging of TtRabD3 provided a stunning illustration of transport over 40–50µM between late phagosomes, in the extreme posterior of the cell, and the cell anterior (Figure 2F, Video S5). Inferring from the EM studies in *Paramecium*, this transport is likely to involve endosomes generated during membrane retrieval following phagosome egestion, which are transported to the oral apparatus. The localization of RabsD17 and D3 suggests the possibility that some Rabs could contribute to coherence within a multi-step pathway by functioning at both early and late steps. Finally, one Rab is localized at the cytoproct itself (Figure 2I).

### Endocytosis is associated with expansions in both conserved and divergent Rabs

*Tetrahymena* has a classical endocytic pathway involving clathrin- and dynamin-dependent formation of small endocytic vesicles, which arise from hundreds of depressions at the plasma membrane called parasomal sacs [40]. These structures occur at regularly-spaced sites along well-defined ciliary rows, also called 1° meridians (Figure 1A and 1B) [41]. The endosomes arising from parasomal sacs can be labeled with the styryl dye FM1-43 [42]. There may also be other pathways of uptake from the plasma membrane, as revealed by residual FM1-43 uptake under conditions of clathrin or dynamin inhibition [42], but this has not been rigorously examined.

**Figure 3:**
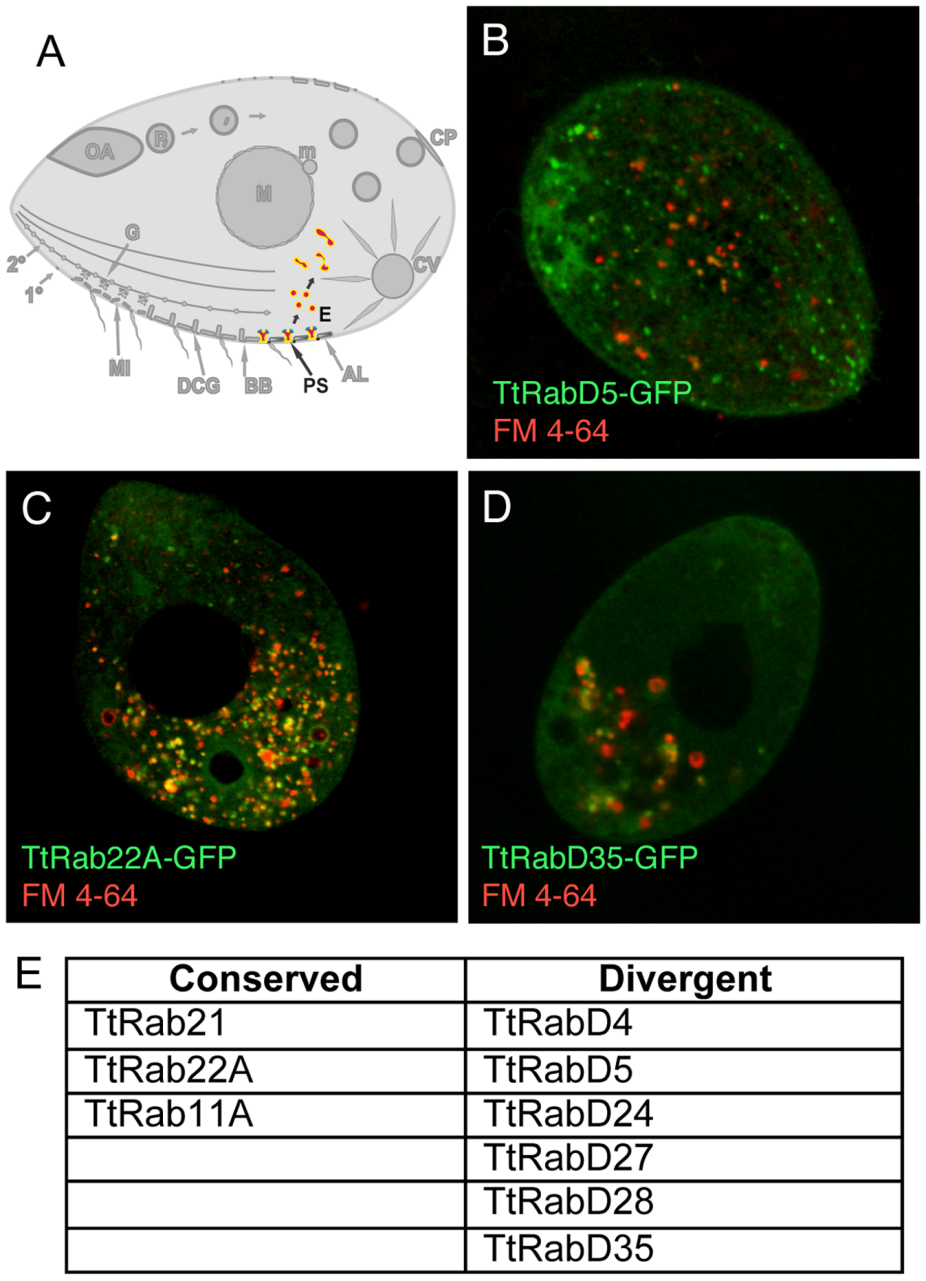
Rabs associated with endocytosis. A. Clathrin-mediated vesicle formation at parasomal sacs (PS) generates endocytic vesicles (E) that coalesce in clumps of tubulovesicular structures at the cell posterior, which may function as recycling endosomes. FM4-64 (red) functions as an endocytic tracer. All panels are confocal images of live cells following induction of GFP-Rab expression for 2 hours in S media, unless otherwise indicated. B. TtRabD5 localizes to parasomal sacs. C. TtRab22A localizes to small abundant cytoplasmic vesicles. D. TtRabD35 localizes to large endosomes toward the cell posterior. E. The full set of TtRabs associated with endocytosis.

We identified endosome-associated GFP-Rabs as those Rabs showing significant colocalization with the styryl dye FM4-64, after first confirming that FM4-64 labels the identical compartments as FM1-43 (Figure S5A). In total, nine of the Rabs in Tetrahymena co-localized with FM4-64 (examples in Figure 3; full set in Figure S6). Only a subset of these Rabs were related to endocytic or endocytic/recycling Rabs from other organisms (Figure 3E). The Rabs show a variable level of overlap with the endocytic tracer, consistent with the idea that multiple, sequential compartments are labeled (Figure S5C). Moreover, the endocytic Rabs can be divided into three broad classes. The 1^st^ group, a single Rab, labels what are likely to be parasomal sacs (Figure 3B). The 2^nd^ group labels small vesicles that are widely distributed throughout the cell (Figure 3C). The 3^rd^ group labels larger vesicles or tubular structures that are concentrated in the cell posterior, often in clumps. Based on previous studies, these are likely to represent a compartment to which FM1-43 gains access several minutes post internalization, and which may function as recycling endosomes (Figure 3D).

### A relatively small group of Rabs appears associated with the outbound secretory pathway

**Figure 4:**
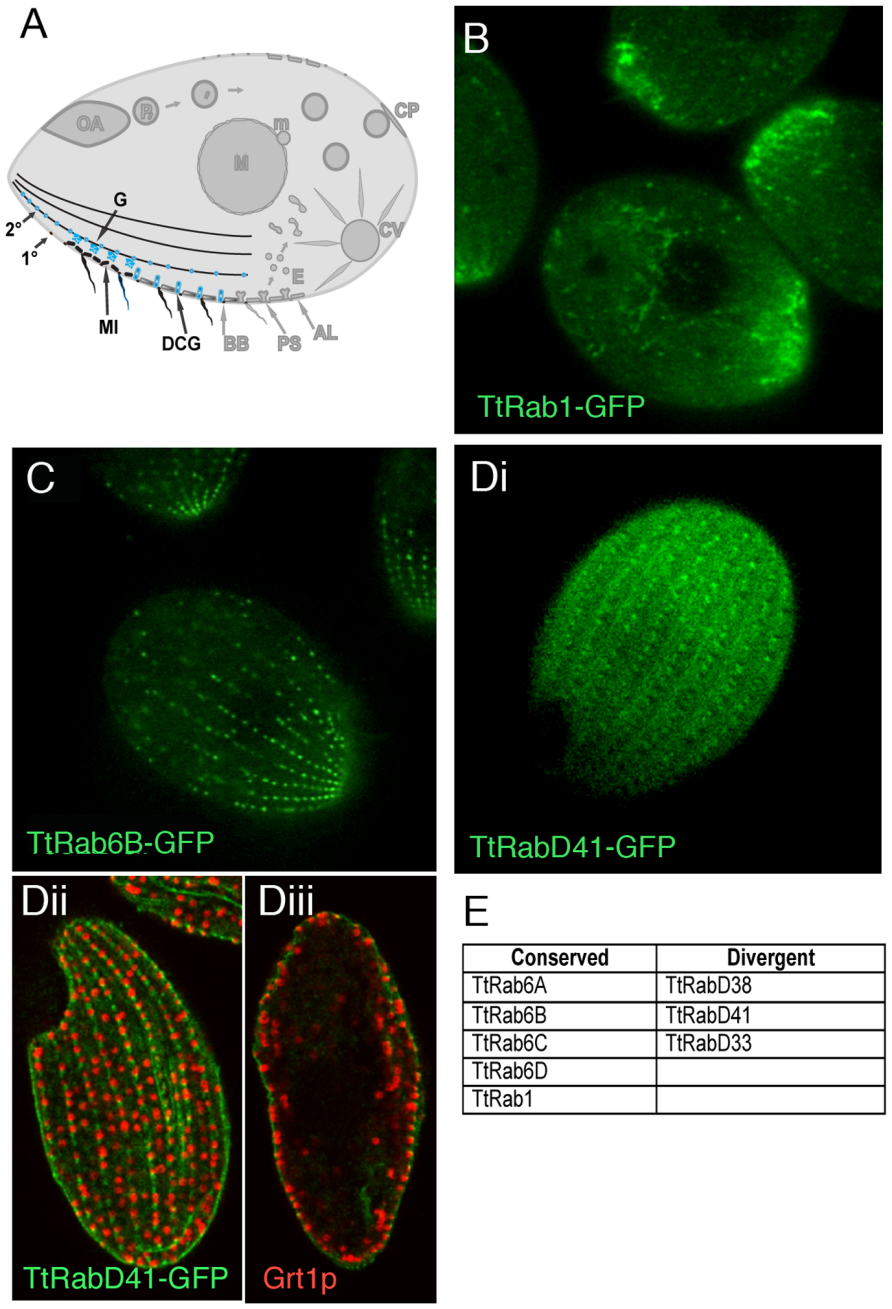
Rabs associated with secretion. A. Known compartments in the secretory pathway. The endoplasmic reticulum in *Tetrahymena* may also include vesicular forms [32] (not shown). Golgi (G) are present as many individual cisterna or short stacks near mitochondria (MI) at the cell periphery. The most prominent secretory vesicles are dense core granules (DCGs), which after maturing in a post-Golgi pathway are transported to stable docking sites along 1° and 2° meridians, where they undergo exocytosis upon extracellular stimulation. B–D. All panels are confocal images of live cells following induction of GFP-Rab expression for 2 hours in S media, unless otherwise indicated. B. TtRab1 is predicted to function in ER-to-Golgi traffic. C. TtRab6B shows the expected distribution for a Golgi-localized Rab. Di. TtRabD41 localizes to puncta at 1° and 2° meridians. Dii and Diii. (Confocal slices of fixed, immunostained cells) TtRabD41 (green) shows extensive colocalization with a DCG marker, Grt1p (red). Dii: optical slice of cell surface; Diii: optical slice through cell midbody. E. The full set of TtRabs associated with secretion.

*Tetrahymena* secrete newly synthesized proteins via at least 3 recognized pathways beginning with translocation into the endoplasmic reticulum (ER). The *Tetrahymena* ER is present as both a cortical as well as cytoplasmic reticulum, while the Golgi are present in multiple copies as small stacks of cisterna that are adjacent to mitochondria, close to the cell periphery [43] (Figure 4A). The 3 known secretory pathways are functionally equivalent to known pathways in mammalian cells: a pathway of rapid constitutive secretion, a pathway of secretion via exocytic fusion of lysosomes, and regulated exocytosis from dense core granule-like bodies called mucocysts. However, the only exocytic sites or secretory carriers that have been directly visualized in *Tetrahymena* are the dense core granules, which dock and undergo exocytic fusion along both the 1° and intervening 2° meridians, at junctions between adjacent alveoli [44].

*Tetrahymena* has a single, very highly expressed member of the Rab1 clade, associated with ER-to-Golgi traffic. This association is robust in Bayesian and Neighbor Joining trees, but not Maximum Likelihood (Figure S9). TtRab1 shows a complex localization pattern, labeling mobile puncta that are primarily concentrated at the anterior end of the cell, and tubular or reticular structures both in the anterior and posterior of the cell (Figure 4B). A 2^nd^, divergent Rab shows similar localization (Figure S7B).

Five Rabs, four of which belong to a conserved Golgi-associated clade, appear to localize to large puncta in a loose meridional array expected for Golgi stacks, and similar to that of a putative Golgi marker, Cda12p [45] (Figure 4C; Figure S7C, S7D, S7E; Video S7). Also consistent with the known location of Golgi, these puncta are positioned near but not at the cell periphery.

TtRabD41 localizes to regularly-spaced puncta at both 1° and 2° meridians (Figure 4Di) and appears to co-localize with the secretory granule marker Grt1p [46], in a pattern suggesting that this Rab may localize to granule docking sites (Figure 4Dii and 4Diii). Since the Rab is also present at cortical sites between granules, it may also localize to unoccupied docking sites.

### A divergent set of Rabs is associated with a specialized organelle, the contractile vacuole

**Figure 5:**
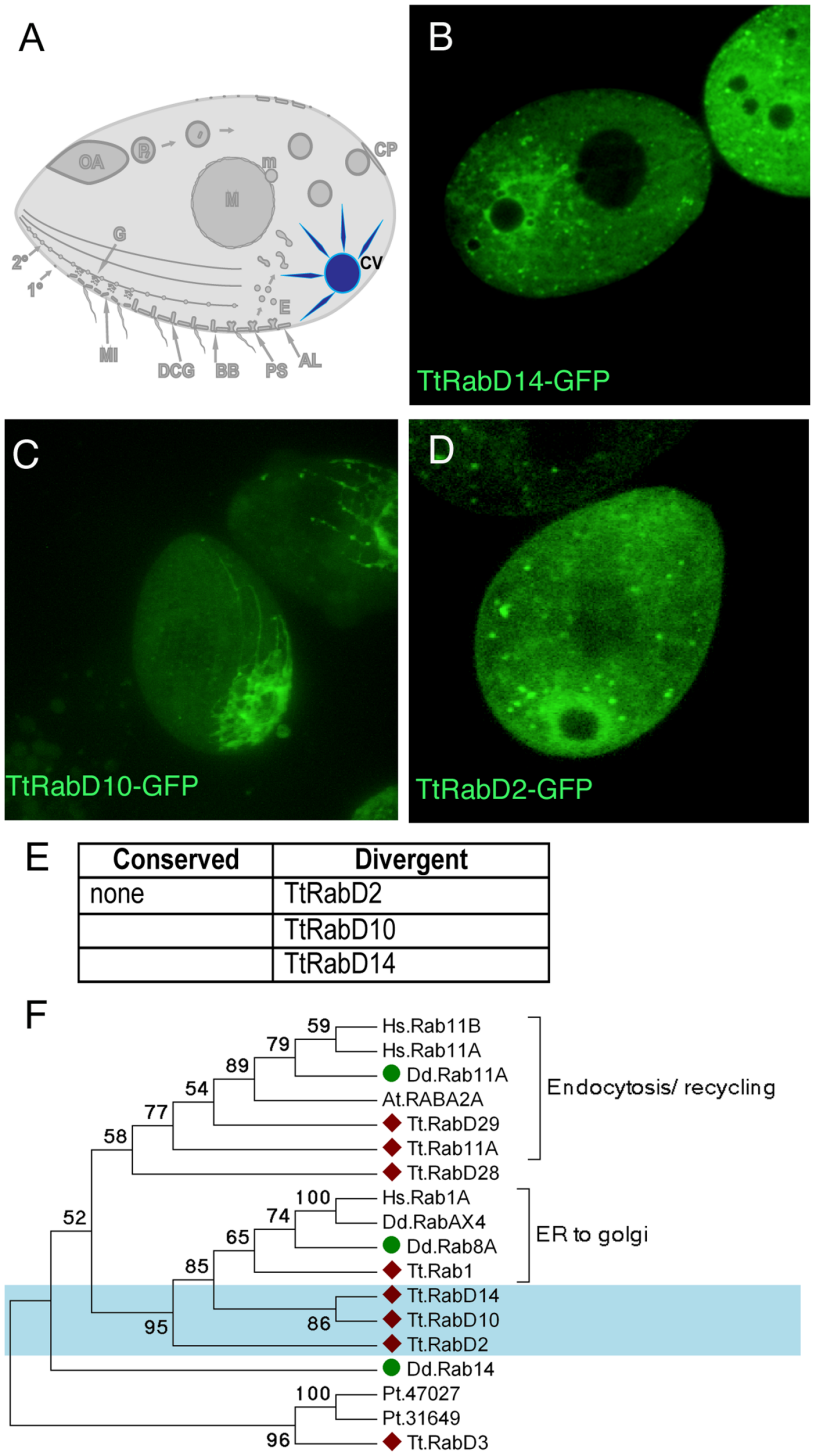
Rabs associated with the contractile vacuole. A. The contractile vacuole (CV) collects water through a set of radial arms that connect to a central bladder, from which water is pumped to the cell exterior during periodic contractions, via pores (not shown) on the cell cortex. B–D. All panels are confocal images of live cells following induction of GFP-Rab expression for 2 hours, unless otherwise indicated. Three Rabs, each with a distinct distribution, are primarily localized to the contractile vacuole. E. The full set of TtRabs associated primarily with the contractile vacuole; other Rabs showing 2° localization to the contractile vacuole are listed in Table S2. F. Maximum likelihood tree showing the relationship between the *Tetrahymena* contractile vacuole Rabs, 3 *Dictyostelium* contractile vacuole Rabs [79], and other *Tetrahymena* Rabs that align with the *Dictyostelium* contractile vacuole Rabs. *Tetrahymena* Rabs are labeled with diamonds; *Tetrahymena* contractile vacuole Rabs are shaded blue; *Dictyostelium* contractile vacuole Rabs are labeled with green circles. In all cases, the *Tetrahymena* Rabs most closely related to *Dictyostelium* contractile vacuole Rabs are not themselves associated with the contractile vacuole.

The contractile vacuole functions to collect water from the cytoplasm and pump it to the cell exterior, to maintain osmotic balance. The complex structure includes a contractile bladder, from which water-collecting tubules extend into the cytoplasm (Figure 5A) [47]. Three divergent and unrelated *Tetrahymena* Rabs primarily labeled the contractile vacuole (Figure 5F). TtRabD14 labels large vesicles that are clearly associated with the contractile vacuole but distinct from the central bladder, while TtRabD10 labels tubular extensions thereof (Figure 5B and 5C). TtRabD2 shows diffuse labeling always centered on the contractile vacuole (Figure 5D). In addition, several other GFP-Rabs show secondary contractile vacuole localization (Table S2).

Active water pumping is required for all cells that lack cell walls and inhabit fresh water, and contractile vacuoles are accordingly found in diverse unicellular lineages. In *Dictyostelium discoideum*, a slime mold very distantly related from Ciliates, the Rabs associated with the contractile vacuole have been described [48] (and refs therein). Based on phylogenetic analysis, none of the *Tetrahymena* contractile vacuole Rabs appears orthologous to any of the functionally related *Dictyostelium* Rabs (Figure 5F).

The remaining *Tetrahymena* Rabs, including proteins that localize to the nuclear envelope, cell cortex, ciliary basal bodies, and numerous other structures, are illustrated in Figure S8.

### *Tetrahymena* Rab phylogeny helps to elucidate the origins of membrane traffic

**Figure 6:**
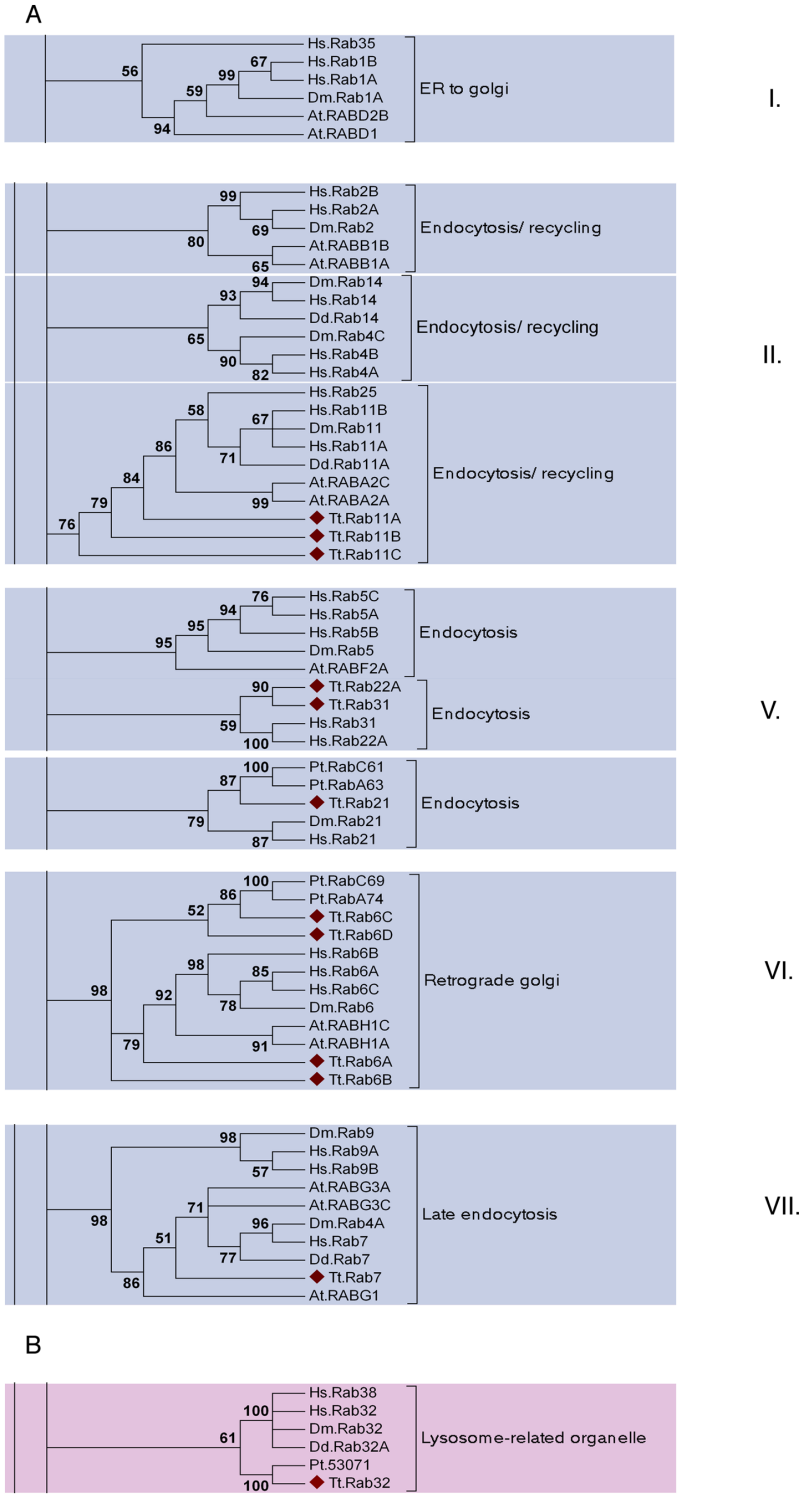
A subset of the TtRabs is highly conserved. Fifteen (27%) of the *Tetrahymena* Rabs align within highly-conserved clades, together with Rabs of known function in other lineages. Shown are panels from the Maximum Likelihood tree in Figure S9, highlighting the 6 clades containing 12 of the conserved *Tetrahymena* Rabs (marked with diamonds). Three additional Rabs were judged to be conserved because they aligned within these conserved clades using both Neighbor-Joining and Bayesian methods (Figure S9): they are TtRab1 (ER-to-Golgi), TtRab4A and TtRab4B (Endocytosis/Recycling). A. Five of the 6 clades (ER to golgi, endocytosis/recycling, endocytosis, retrograde golgi and late endocytosis) belong to a group of 8 Rab clades that have been identified as a core group that is broadly conserved among eukaryotes. The core groups have been numbered following [11]. B. The sixth conserved clade, not previously considered as a core group, contains Rabs in Opisthokonts (*H. sapiens*, *D. melanogaster*), Amoebozoans (*Dictyostelium discoideum*) and Chromalveolates (*Tetrahymena thermophila* and *Paramecium tetraurelia*). Animal Rabs in this clade are associated with lysosome-related organelles [80], while the *Dictyostelium* Rab has not been functionally characterized.

We used maximum likelihood, Bayesian, and neighbor-joining methods to understand the relatedness of the *Tetrahymena* Rabs to those in other lineages (Figure S9). Some of the bootstrap values for nodes linking the Ciliate sequences with those of other lineages were low, as expected in light of the known deep evolutionary divergence between the Alveolates (Ciliates, Dinoflagellates, Apicomplexans) and other lineages [49]. Nonetheless, these reconstructions produced strong evidence that 15 of the *Tetrahymena* Rabs align in clades with Rabs in animals and other lineages, and can therefore be considered as highly conserved. The conserved *Tetrahymena* Rabs fall within six predicted functional groups (Figure 6). Five of these clades were previously defined, by comparison of many eukaryotic genomes, to belong to a core set of eight whose wide distribution in existing eukaryotes implies that all may have been present in the last common eukaryotic ancestor [11]. The *Tetrahymena* Rabs in this group are predicted, based on sequence similarity, to be associated with ER-to-golgi traffic (intermediate compartment) (1 Rab), endocytosis (3 Rabs), endocytic recycling (5 Rabs), late endocytosis (1 Rab), and retrograde golgi traffic (4 Rabs). *Tetrahymena* does not appear to have representatives within three remaining core Rab clades. Two of these, including human Rabs 8,10,13, 19 and 30, are associated with Golgi traffic. The third clade is associated with regulated exocytosis, and includes human Rabs 3 and 27.

Strikingly, a single *Tetrahymena* Rab does not belong to the previously defined core set, but nonetheless aligns with a clade with representatives in both Opisthokonts (*H. sapiens*, *D. melanogaster*) and Amoebozoa (*D. discoideum*) (Figure 6B). The animal Rabs in this group, which are all associated with transport of lysosome-related organelles, all share a WDIAGQE motif, which is also found in both the *Dictyostelium* and *Tetrahymena* members but not in Rabs from any other clade [50]. We propose that this clade, with representatives from at least three deeply divergent lineages, represents a previously unrecognized core Rab clade in eukaryotes.

### Sequence-conserved Rabs, while generally being associated with the predicted pathways, show significant exceptions that may reveal new pathways

One feature that emerged from comparison of phylogenetic assignments and localization data was that some highly conserved Rabs failed to show clear association with the expected compartments. In particular, four of the putative endosomal Rabs showed little or no colocalization with FM4-64. Three of these were localized to structures associated with the phagocytic pathway. Two of these, 11B and 4B, labeled highly mobile puncta at the oral apparatus, whose lack of overlap with FM 4-64 indicates that these are not vesicles pinching off from the plasma membrane (Figure S4A, S4D). This is graphically reinforced by time-lapse movies of cells expressing TtRab11B-GFP, which show that labeled puncta, which are likely to be vesicles, are traveling *toward* the oral apparatus along a cytoplasmic fiber (Video S6), where they may be delivering bulk membrane required for phagocytosis [39]. TtRab4A labels vesicles that accumulate near the cytoproct, which is a zone where end-stage phagosomes fuse and eject undigested contents [51]. The fourth Rab in this group, TtRab31, localized to puncta along cortical meridians (Figure S8A).

## Discussion

While the initial annotation of the *Tetrahymena* macronuclear genome provided a rough sketch of the genetic underpinnings of cellular complexity, our focused analysis of a single gene family illuminates the pathways of membrane traffic in these cells, including their dynamics and evolutionary origins. *T. thermophila* encodes at least 56 Rabs and 17 Rab-like proteins, the latter lacking the residues required for prenylation [52]. Additional *Tetrahymena* Rabs may have escaped detection if extreme sequence divergence precluded their detection by our homology-based approach or if those genes were present on remaining gaps in the sequenced genome, although EST-based evidence suggests that few highly expressed genes can be present in such gaps [53].

More than 90% of the *Tetrahymena* Rabs are expressed under typical growth conditions. This suggests that the size of the gene family reflects the need to specify a large set of concurrent steps, assuming that most Rabs serve unique functions. While four conserved Rabs showed similar expression patterns as well as localization to putative Golgi, few other Rabs appeared to share identical localization patterns, and even Rabs associated with a single structure often showed subtle differences in distribution. These observations are also consistent with the high degree of sequence divergence between family members. For example, with the exception of a single pair of 84% identical Rabs that may be related by gene conversion (TtRabD21 and TtRabD22), there are few sequence-similar Rabs in *Tetrahymena*. Moreover, even pairs such as Rabs D5 and D17, which are 70% sequence identical, show clearly distinct patterns of localization as well as expression.

Roughly 27% of the *Tetrahymena* Rabs aligned with orthologs in other eukaryotic lineages. The modest fraction may reflect a relatively fast rate of evolution of genes in Ciliates relative to other organisms [54], potentially compounded by limitations on phylogenetic analysis due to the paucity of sequenced ciliate genomes and the absence of any sequenced genome from the sister Dinoflagellate clade. However, non-Ciliate protists with large Rab cohorts show a similar preponderance of divergent Rabs: 51/65 in *T. vaginalis*, and 69/91 in *E. histolytica* [14], [15]. In comparison, the paralogous radiation within Rabs of animals and plants resulted primarily in expansions of conserved Rab clades: 35 of 60 *H. sapiens* Rabs are conserved, as are 48 of 57 in *A. thaliana* [55]. These disparities may suggest that multicellularity, when it arose in a lineage, imposed subsequent constraints on the evolution of intracellular membrane traffic.

Using Rab sequences mined from divergent eukaryotic genomes, it was deduced that an ancestral eukaryotic cell contained Rabs corresponding to eight core pathways [11]. We used phylogenetic analysis to gauge the allotment of the 15 sequence-conserved *Tetrahymena* Rabs to these pathways. Surprisingly, one of the conserved Rabs did not fall into any of the proposed core clades, but nonetheless aligned with Rabs in both animals and *Dictyostelium*, with which it also shared a unique motif [50]. The animal Rabs in this clade are associated with transport of lysosome-related organelles, while the *Tetrahymena* protein localized to phagosomes. Because the related *Dictyostelium* Rab has not been characterized, there are not yet sufficient data to infer an ancestral function for what we propose to be a deeply conserved clade. The remaining 14 conserved Rabs in *Tetrahymena* fall within five of the proposed core pathways [11]: ER-to-golgi (I),endocytosis/recycling (II), endocytosis (III), retrograde golgi (VI) and late endocytosis (VII). The localization data suggest that paralogs within the endocytic and endocytic/recycling clades are likely to reflect multiple specialized pathways, while the paralogs within the retrograde Golgi clade may potentially reflect a dosage requirement. The idea that endocytic pathways have undergone greater expansion and diversification over evolutionary time, compared with some other pathways, was also inferred from SNARE phylogeny [56],[57]. *Tetrahymena* appear to have lost the core clades corresponding to golgi-related (IV and VIII), and regulated exocytosis (III) pathways. Such lineage-restricted loss of specific core Rabs has previously been noted, for example in fungi [58]. Since *Tetrahymena* has a prominent pathway of stimulus-dependent protein release, the absence of Rabs in the conserved regulated exocytosis clade is consistent with an independent origin of secretory granules in ciliates [59]. *Tetrahymena* lacks an ortholog of human Rab8, which is required for ciliogenesis [60]. A similar function may be played by a divergent Rab in ciliates, although we did not identify such a Rab in this survey.

### Dynamic flexibility of Rab GTPases

Growing vs. starved *Tetrahymena* differ in pathways involved in feeding and secretion, while conjugation brings pronounced changes to the cell cortex, and to nuclear and other organelles [22]. We found that membrane traffic in starvation and conjugation chiefly involve the same set of Rabs that are expressed in growing cells. However, several Rabs are exclusively expressed in growth and/or starvation, some showing dramatic expression peaks at distinct stages.

**Figure 7:**
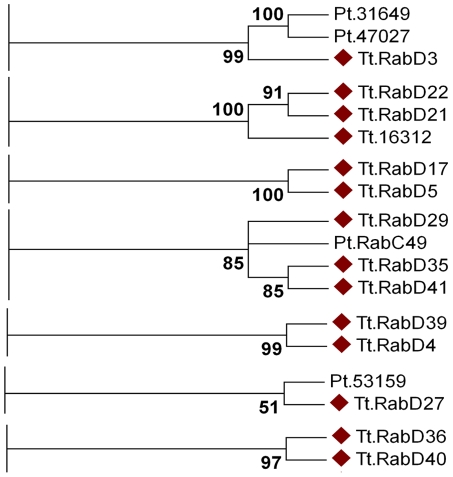
At least fourteen *Tetrahymena* Rabs appear lineage-restricted. Shown are panels from the Maximum likelihood tree in Figure S9, highlighting the 7 clades containing lineage-specific *Tetrahymena* Rabs (marked with diamonds). Three clades also contain *Paramecium* Rabs.

To understand the contributions of Rabs to cellular plasticity on an evolutionary timescale, one pertinent question is whether specific subsets of Rabs arose in Ciliates. Among the non-conserved Rabs, a subset of 14 aligned using all tree-building methods into seven clades containing only other Ciliate genes (Figure 7). Four of these clades are entirely composed of *T. thermophila* genes, suggesting four relatively recent paralogous expansions. In contrast, the remaining *T. thermophila* Rabs aligned in 3 clades with Rabs in *Paramecium tetraurelia*. These 14 Rabs may tentatively be considered as lineage-restricted. One interesting question is whether Ciliate-restricted Rabs are preferentially associated with pathways that arose in this lineage. Four of the currently recognized lineage-restricted Rabs localize to the oral apparatus, contractile vacuole, or cytoproct, which are structures that are likely to have undergone extensive elaboration, at a minimum, in the Ciliate lineage. The remainder localize to endosomes, the cell cortex, phagosomes, and as-yet undefined structures.

The dataset also allowed us to ask whether structures or pathways that appear deeply conserved have conserved Rab determinants. In humans, 18 Rabs are associated with phagosomes, while 11 *Tetrahymena* Rabs were found on phagosomes. Only two of these *Tetrahymena* Rabs are orthologous to those in animals, TtRabs 32 and 7 (Figure 2J). TtRab32 belongs to the Rab32/38 clade, whose members in animals are most strongly linked with transport of lysosome-related organelles. Similarly, Rab7 in both humans and *Tetrahymena* is associated with lysosomes as well as phagosomes. Therefore, most phagosomal Rabs in humans and *Tetrahymena* are unrelated, and the related Rabs are primarily associated with lysosomes. This implies either that mechanisms of phagosome maturation arose independently in animals and ciliates, or that this set of Rabs evolved under relatively few constraints in one or both lineages. In this regard, it is noteworthy that some phagosomal Rabs may have arisen within ciliates. The inference that phagosomes in *Tetrahymena* may have evolved separately from those in animals is consistent with the finding that phagosome-associated syntaxins in another Ciliate, *Paramecium tetraurelia*, form a lineage-restricted clade [61]. Similarly, we found no relatedness between Rabs associated with the contractile vacuole in *Tetrahymena* and in *Dictyostelium*. These results suggest that, in some cases, the similar cellular structures or pathways in different lineages do not primarily reflect constraints on an inherited ancestral pathway but rather parallel selective pressures leading via innovation to similar outcomes. This would depend, in the context of this paper, on Rabs that evolve novel functions, of which there are several good illustrations in the *Tetrahymena* cohort. First, several phylogenetically conserved Rabs were not associated with the predicted compartments, a finding that also underscores the importance of doing phylogenetic and localization analysis in parallel. Secondly, there is evidence for rapid Rab evolution within the lineage-restricted Rabs. Some lineage-restricted Rabs appear to have retained similar functions, judging by the cortical localization of TtRabs D41, D29 and D35. In contrast, TtRabs D39 and D4 are nearest neighbors in a single clade, but the former localizes to the cytoproct and oral primordium while the latter is found at endosomes.

In summary, analysis of the large Rab gene family in *Tetrahymena* has provided an extensive set of new molecular markers for studies in this organism, and has provided insights into cellular and evolutionary aspects of membrane plasticity. Though Ciliate genes may be prone to undergoing fast evolution [54], the observation that Ciliate and non-Ciliate protists show comparable ratios of conserved to divergent Rabs suggests that many of our observations are generalizable. This work sets the stage for functional analysis of informative Rab family members in this organism. More broadly, Rabs belong to a small set of proteins that, as products of large gene families, act as compartmental determinants of membrane traffic. The availability of sequenced genomes from divergent lineages highlights the need for combined approaches such as those taken here, to understand the consequences of gene family expansions and the evolutionary flexibility that is built into fundamental cell biological features, such as the complex network of membrane trafficking pathways that are crucial for homeostasis and signaling.

## Materials and Methods

### Rab identification and cloning

Beginning with amino acid sequences of known human Rabs (accession numbers in [11]), we previously identified 70 putative *T. thermophila* Rabs by tblastn searches of the Macronuclear (Mac) genome, using the primary *Tetrahymena* hits as queries to detect additional *Tetrahymena* Rabs [23]. We subsequently annotated all genes using ESTs corresponding to 45 Tetrahymena Rabs for which ESTs became available during genome refinement [53], aligning the ESTs with genomic sequences as well as the predicted mRNAs using Muscle (**MU**ltiple **S**equence **C**omparison by **L**og-**E**xpectation: http://www.ebi.ac.uk/Tools/muscle/index.html), and viewing the results using Seaview (http://pbil.univ-lyon1.fr/software/seaview.html). Confirmed mRNA sequences were translated (including 5′ and 3′ UTRs) in 3 frames to establish the ORF encoding the conserved Rab motifs and predict the start sites, which in general lay shortly upstream of the 1^st^ conserved motif. Stop codons were identified as the 1^st^ in-frame UGA (the sole stop codon used in *T. thermophila* [62]. In this process, we identified 3 Rabs not detected in earlier work, adjusted several gene predictions, and disqualified 17 previously identified Rabs that were determined to be Rab-like. Forward and reverse primers (Table S3) were designed to initiate from start and stop codons. All Rab genes were PCR amplified from genomic DNA isolated by phenol-chloroform extraction from strain CU428.1, using Pfu Ultra polymerase (Agilent Technologies, Santa Clara, CA), and confirmed by sequencing. GenBank ID numbers of all genes are listed in Table S1.

### Phylogenetic tree-building methods

We used three different tree-building methods (maximum likelihood [63], Bayesian [64], [65] and neighbor-joining [66]) including all predicted Rabs from *T. thermophila* and *H. sapiens* and selected Rabs from *D. melanogaster*, *A. thaliana*, *D. discoideum* and *P. tetraurelia*. The basic tree topology presented in this manuscript is supported by all three distinct algorithms. In cases where detailed topological conclusions are supported by 2 out of 3 approaches, this is specified in the text. Protein sequences were aligned using Muscle [67] (http://www.ebi.ac.uk/Tools/muscle/index.html), and gaps and the C-terminal hypervariable region of each Rab (∼30–80 residues) were manually removed from the multiple alignment using Seaview [68] (http://pbil.univ-lyon1.fr/software/seaview.html). The output from alignment and gap removal were 167 total aligned sites, which were then used as input for bootstrap analysis using Seqboot from Phylip [69] (http://evolution.genetics.washington.edu/phylip.html). 100 bootstraps were run. The bootstrapped outfile was used as the input for a maximum likelihood test using the Phyml program (http://atgc.lirmm.fr/phyml/), using the WAG substitution model. For Bayesian analysis, the bootstrapped outfile from Seqboot was used (in Nexus format) to run an analysis using MrBayes (http://mrbayes.csit.fsu.edu/) for 100,000 generations, with sampling every 100 generations.

For *Tetrahymena* Rabs that failed to align with any Rabs in *H. sapiens*, *S. cerevisiae*, or *A. thaliana*, we sought orthologs from other species using tblastn. The top-scoring putative homologs were then used in reverse BLAST searches to screen the *Tetrahymena* genome. If the original *Tetrahymena* Rab gene was the top-scoring hit in this reciprocal BLAST, the genes were considered potential orthologs and added to the large phylogenetic tree analysis. The chief outcome of this approach was addition of *Paramecium* genes to the phylogenies.

### Rab nomenclature

Conserved Rabs were named according to their human ortholog. Divergent Rabs were numbered D1–D41.

### Transcription profiling

The expression profiles of all *Tetrahymena* Rabs in growing, starved and conjugating cultures were downloaded from the whole genome expression database at http://tged.ihb.ac.cn/ [27].

### Expression of Rab-GFPs

The Gateway (Invitrogen) system was used to engineer each Rab as a GFP (Green Fluorescent Protein) fusion. First, PCR-amplified Rab genes were TOPO cloned (Invitrogen) into the pENTR-D-TOPO entry vector. CACC was added to each forward primer in order to allow directional cloning into pENTR-D. The pENTR clones were sequenced and the genes recombined using the Clonase reaction into the target Gateway-based *T. thermophila* expression vector pIGF-GTW, a gift from Doug Chalker [70]. We used site-specific mutagenesis to change the GFP gene in pIGF-GTW to the monomeric variant (A206K) [71]. Genes subcloned into pIGF-GTW are fused at their C-terminus, via a linker sequence, to the GFP gene, with the fusion under the transcriptional control of the cadmium-inducible *MTT1* promoter [72]. When introduced into *Tetrahymena*, the vector is maintained as an extrachromosomal Mac plasmid and confers paromomycin resistance. TtRabD37 could not be cloned into pENTR; in addition, TtRab11C could not be expressed in *Tetrahymena* as a protein of the predicted size.

### Cell culture and transformation

*T. thermophila* strains were provided by Peter Bruns and Donna Cassidy-Hanley (Cornell University)(CU428.1) and Eduardo Orias (UC Santa Barbara)(B2086). Cells were grown at 30°C in SPP media (1% proteose peptone, 0.2% dextrose, 0.1% yeast extract, 0.009% ferric EDTA) with shaking. To reduce autofluorescence background in food vacuoles when imaging cells expressing GFP-tagged proteins, the cells were transferred to S medium (0.2% yeast extract plus 0.003% iron EDTA) for 2h prior to imaging. For experiments requiring starvation (also at 30°), cells were transferred to DMC, a one-tenth dilution of Dryl's (1.7 mM sodium citrate, 1mM NaH_2_PO_4_, 1mM Na_2_HPO_4_, 0.5 mM CaCl_2_) supplemented with an additional 0.1 mM MgCl_2_ and 0.5 mM CaCl_2_.

Transformation of GFP fusions was by conjugant electroporation [73]. Briefly, *T. thermophila* strains CU428 and B2086, after 10h of conjugation, were combined with 20µg of DNA and electroporated; after 1d 100 µg/ml paromomycin sulfate was added to select for transformants, which were picked at 5 d. In general, the transformants were maintained at room temperature by weekly transfer in 24-well plates in SPP with paromomycin, and showed stable expression of the Rab-GFP for at least 6 weeks and often longer, depending upon the particular Rab. Transformants were stored in parallel in tube cultures (in 2% proteose peptone, at room temperature, no paromomycin), and showed stable Rab-GFP expression for 2–3 months.

### Live cell microscopy

The GFP-transgenes were induced with the lowest level of CdCl_2_ (0.25–1µg/ml, determined empirically for each strain) that produced a discernible localization pattern. We analyzed 4 independent lines per transformation. The rare cases where differences were seen were due to variation in levels of what appeared to be diffuse cytoplasmic fluorescence. For imaging, transformants were grown overnight in SPP media and then transferred, 2h before imaging, to S medium at room temperature containing 0.25–1µg/ml CdCl_2_. Cells induced in DMC required lower levels of CdCl_2_ (0.1–0.25µg/ml).

Microscopy was performed at room temperature. For initial visualization, live GFP-Rab-expressing *Tetrahymena* cells were imaged on an Olympus DSU spinning disk inverted confocal microscope with a 100× objective. Cells were immobilized by mixing 1∶1 with either 6% polyethylene oxide (PEO: MW = ca.900,000), or 6–10% poly(ethylene glycol)-polyalanine (2000–1500) diblock copolymer solutions that undergo sol-to-gel transition as the temperature increases [74], both made in S media. Images were acquired with an EM-CCD Hamamatsu camera and SlideBook acquisition software. Unless indicated, figures are a projection of a z-stack of the entire cell or the cell mid-section. The Z projections and color channel merges, as well as adjustments to brightness and contrast, were made using the public domain NIH ImageJ program (http://rsbweb.nih.gov/ij/). Each image shown is representative of the majority of cells expressing that Rab-GFP. A fraction of the cells appeared to contain multivesicular phagosomes, which accumulated FM4-64 in cells exposed to this endocytic tracer (see below) and may be a response to stress. This fraction increased with time under the coverslip and was particularly evident in PEO-immobilized samples. Both for microscopy and for quantitative analysis, we chose cells with few or no such structures.

Time-lapse and/or image stack movies of all Rabs, as well as additional images of some Rabs showing colocalization (see below), are accessible at tetrahymenacell.uchicago.edu.

### Labeling endocytic and phagocytic compartments with FM 4-64, India ink, or DsRed-bacteria

*Tetrahymena* transformants were incubated for 5min with 5µM FM 4-64 dye [75] (Invitrogen) (excitation/emission maxima ∼515/640 nm). The cells were then pelleted and resuspended in ∼40µl of supernatant, and immobilized as above for microscopy for up to 2h. In all cases, Rabs that showed colocalization with FM4-64 showed this pattern within 10 min. Cells were imaged on a Leica TCS SP2 AOBS laser scanning confocal microscope with LCS Leica confocal software with simultaneous capture in the green (495–520 nm) and red (656–746 nm) channels. Images shown are single slices for clarity. As above, adjustments in brightness and contrast were conducted in ImageJ. Phagosomes were labeled with india ink by incubating *Tetrahymena* for 1–2 h prior to imaging with 2.5% v/v india ink in S medium. The cells were simultaneously induced with CdCl_2_ (for 2h) to induce transgene expression. Alternatively, phagosomes were labeled by incubating *Tetrahymena* in S medium for 2h with *E. coli* expressing dsRed-Express2 [76] (gift of R. Strack and B. Glick, U. Chicago)(adding 0.5% v/v of an overnight culture), with simultaneous induction of Rab-GFP expression. Cells were imaged using a Zeiss Axioplan2 upright widefield microscope with a Hamamatsu camera and Axiovision software (using a 63× objective).

### Labeling acidic compartments with Lysotracker

Lysosomes and phagosomes were labeled in cells expressing TtRab7-GFP by addition of 0.05% (v/v) LysoTracker (Invitrogen) for 90min to cells in SPP at 22°C, in which Rab-GFP expression was induced for a total of 150min with 0.5µg/ml CdCl_2_. The cells were pelleted and then resuspended in PEO for immobilization and microscopy. Cells were imaged using a Zeiss LSM-510 laser scanning confocal microscope with LSM 5 software.

### Image quantification

Overlap quantification (e.g., a Rab-GFP and FM4-64) was performed using NIH ImageJ, based on the method described in [77]. Briefly, pixels of interest were identified by generating a mask for each channel to eliminate background signal. Single focal plane images were used and we defined a threshold value in red and green channels separately that included only the brightest labeled structures. Overlap was defined as the percentage of total signal ‘intensity’ present in shared pixels. To recover intensity lost in the creation of the masks, we modified the binarized masks using subtract and invert functions in ImageJ to regain the green and/or red intensity values above threshold. To calculate overlap for the green signal we used the ratio of the average intensity of the green pixels in the AND mask over that of the green mask. We did the converse for the red signal. For each line, at least 4 cells were analyzed.

### Immunofluorescence

Secretory granules (mucocysts) were immunostained with the 4D11 or 5E9 monoclonal antibodies as described in [78], except fixation was at room temperature rather than on ice. Basal bodies were stained using the 20H5 anti-centrin antibody (gift of Jeff Salisbury, Mayo Clinic), with Texas Red-conjugated goat anti-mouse 2° antibody (Invitrogen). Immunostained cells were mounted with 0.1µM Trolox to inhibit bleaching and imaged the Leica SP2 laser scanning confocal microscope as described above.

### Cell lysates and western blotting

Whole cell lysates were prepared by 10% trichloroacetic acid precipitation (∼1×10^6^ cells in SPP per sample) and then dissolved in SDS-PAGE sample buffer. For western blots, 2×10^5^ cell equivalents per sample were resolved by SDS-PAGE, transferred to 0.45µM nitrocellulose (General Electric Water and Process Technologies), probed with polyclonal anti-GFP antibody at 1∶1,000 dilution (Invitrogen) and Alexa 680 anti-rabbit antibody (Invitrogen) at 1∶5,000 dilution, and imaged using the Li-Cor Odyssey protocol and scanner (http://www.licor.com).

## Supporting Information

Figure S1 Multiple sequence alignment of all *T. thermophila* Rabs showing the five Rab-defining motifs. The consensus motifs for other classes of Ras-related GTPases are shown. The gaps shown between motifs do not represent the real spacing in the polypeptide sequences.
(1.28 MB TIF)

Figure S2 Example of stage-specific or -enhanced expression. Expression profile is from the *Tetrahymena* Gene Expression Database at http://tged.ihb.ac.cn/. Cell images are of live cells taken with a spinning disk confocal microscope, in S media (B) and DMC starvation media (C). A. TtRabD12 is predicted from expression levels to be stage-specific for starvation. B. TtRabD12-GFP shows indeterminate localization in growing cells. C. In starved cells, the Rab is tightly localized in cytosplasmic puncta.
(0.45 MB TIF)

Figure S3 Expression of GFP-tagged proteins at the predicted sizes was confirmed by Western blotting of protein lysates, prepared as described in Methods. Blots were probed with a polyclonal anti-GFP antibody. SDS-PAGE low range molecular weight standards (Biorad) are labeled at 66 and 45 kD.
Lane 1. TtRabD15; 2. D25; 3. D27; 4. Rab11B; 5. D3; 6. Rab6B; 7. D19; 8. D17; 9. Rab22A; 10. D11; 11. D35; 12. D34; 13. Rab21; 14. D38; 15. D39; 16. Rab6C; 17. Rab6D; 18. D6; 19. Rab4B; 20. D28; 21. D7; 22. D4; 23. D29; 24. D26; 25. D36; 26. Rab6A; 27. Rab4A; 28. D24; 29. Rab7; 30. D16; 31. D14; 32. D30; 33. D5; 34. D20; 35. D21; 36. D40; 37. D18; 38. Rab1; 39. Rab11A; 40. D41; 41. D12; 42. D31; 43. D32; 44. D9; 45. Rab32; 46. D13; 47. D10; 48. D2; 49. D33. Seven Rabs are not shown: RabD37 could not be cloned, Rab11C was not expressed at the predicted size; RabsD22 and D1 were not stable at levels high enough for either visualization or Western blotting; RabsD23 and 31, which when expressed at levels that showed distinct though dim localization signals, could not be detected by Western blot.
(1.97 MB TIF)

Figure S4 The set of Rabs associated with phagocytic uptake or digestion. All panels are confocal images of live cells following induction of GFP-Rab expression for 2 hours in S media, unless otherwise indicated. Green: TtRab-GFP. A–E. Rabs that label the oral apparatus. Additional observations: A. (a projection of a z-stack) TtRab4B labels the oral apparatus, primary meridians at the cortex, and bright vesicles in the posterior cytoplasm. D. TtRab11B labels part of the oral apparatus, including the deep fiber, and small vesicles concentrated in the anterior cytoplasm (movie, Video S6). E. (Projection of maximum intensities of a z stack). TtRabD5 puncta are strikingly mobile, especially near the oral apparatus. This Rab also shows localization to parasomal sacs, and at the contractile vacuole. F–O. Rabs that primarily label some or all phagosomes, visualized by uptake of dsRed-expressing bacteria (red) or india ink (black, shown as overlays with DIC channel. Additional observations: J. TtRabD19 secondarily labels 1° meridians including part of the oral apparatus. L. TtRabD30 labels irregularly spaced puncta at primary cortical meridians, with little or no overlap with a basal body marker. N. In addition to labeling the oral apparatus, TtRabD17 labels phagosomes in the cell posterior, and also small mobile vesicles. O. TtRabD3 localizes to phagosomes in the cell posterior and also to vesicles transported along cytoplasmic microtubules (movie, Video S5) P. TtRabD39 strongly labels the cytoproct (the plasma membrane zone where mature phagosomes fuse to egest undigested contents) and secondarily labels the nascent oral apparatus as well as 1° and 2° meridians. Q,R. For cells expressing TtRab7 in SPP medium (Q), LysoTracker (red) labels both phagosomes (larger red vacuoles) and smaller vesicles (red and yellow) that are likely to be lysosomes. TtRab7 colocalizes with the latter, which are concentrated in the anterior cytoplasm. R. In cells cultured in S medium, TtRab7 co-localizes with LysoTracker at very small puncta at the periphery of phagosomes (arrowhead). In all panels, the *Tetrahymena* cells are ∼50×∼20 µM.
(6.17 MB TIF)

Figure S5 Colocalization of endocytic Rabs with lipophilic styryl dyes. A. Cells incubated for 60 min with FM 1-43 (green; shown previously to be an accurate endocytic tracer in *Tetrahymena* [42] and FM 4-64 (red) show near-complete overlap between the two dyes. B. The individual green and red channels, and the merge, shown for TtRab11A. C. Colocalization of FM 4-64 and GFP in cells expressing endocytic Rab-GFPs, quantified as described in Methods. For each line, each data point represents colocalization measured in a single cell. Complete overlap would register as 1.0 (on the ordinate).
(0.93 MB TIF)

Figure S6 The set of Rabs associated with endocytosis. All panels are confocal images of live cells following induction of GFP-Rab expression for 2 hours in S media, unless otherwise indicated. Rab-GFP expression is in the green channel; FM4-64 is in the red channel. Images are single confocal slices. Additional observations: A. TtRabD5 also labels the base of the oral apparatus (see Figure S4). C. TtRab21 co-localizes with small FM 4-64-positive vesicles, including at the cortex, but not with larger FM 4-64-positive cytoplasmic vesicles. It also shows diffuse localization near the cortex and the contractile vacuole. D. TtRabD28 also labels the contractile vacuole. E. TtRabD4 labels some cortical puncta. G. TtRabD24 is exclusively cytoplasmic with no cortical signal. H. TtRab11A labels both vesicular and tubular endosomes. This Rab also appears concentrated in a zone around the contractile vacuole, and to a lesser extent at the plasma membrane and in the anterior cytoplasm. Panels Ii–Iiii represent the red, green and merged channels respectively for a TtRabD35-GFP expressing cell. This Rab also labels the cortex at the anterior end of the cell (see Figure S8).
(3.08 MB TIF)

Figure S7 The set of Rabs associated with protein secretion. All panels are confocal images of live cells following induction of GFP-Rab expression for 2 hours in S media, unless otherwise indicated. A–B. TtRabs putatively associated with the ER or ER-to-Golgi traffic. C–G. TtRabs putatively associated with the Golgi. C,E,F are projections of z stack maximum intensities, while D is a single slice taken at the labeled meridians. All five Rabs label mobile puncta that are near but not at the cortex, which are brighter and more concentrated at the anterior end, as well as mobile puncta within in the cytoplasm (Video S7). Hi–Hiii. TtRabD41 shows extensive colocalization with docked dense core granules, for which Grt1p is a marker.
(2.49 MB TIF)

Figure S8 Fourteen TtRabs localize to the cell cortex, the macronuclear envelope, and other cell structures. All panels are confocal images of live cells following induction of GFP-Rab expression for 2 hours in S media, unless otherwise indicated. A. TtRab31 localization was limited to faintly fluorescent puncta at primary meridians. Because of the long exposure times needed to capture this signal, phagosomes in these cells are illuminated by autofluorescence. Shown is a projection of a z stack. B. TtRabD23 has a localization pattern that is very similar to basal bodies. C. TtRabD36, in addition to showing continuous labeling of 1° meridians, labels mobile cytoplasmic puncta. D. TtRabD40 appears to continuously label 1° meridians, and also labels bright puncta both at meridians and in the cytoplasm. E. TtRabD18 labels the cortex uniformly, but in many cells also shows a small number of bright cortical puncta and dim fluorescent cytoplasmic puncta. F. TtRabD29 shows uniform labeling of the cortex. G. TtRabD31 labels the macronuclear envelope brightly, and shows faint puncta along 1° meridians. H. TtRab4A brightly labels medium-sized puncta toward the posterior end of 1° meridians, which are strikingly concentrated near the cytoproct. It also labels the oral apparatus and small puncta along 1° meridians. Only the puncta near the cytoproct are mobile. I. TtRabD6 labels heterogeneous mobile cytoplasmic vesicles that show little or no overlap with FM4-64, and that move rapidly and directionally; some cells show labeled mobile tubules that extend along meridians or from the cortex into the cytoplasm. J. TtRabD7 labels heterogeneous vesicles that do not accumulate FM 4-64 and are concentrated in the cell posterior, with some labeled vesicles appearing to move along microtubule tracks. K. (Whole cell z projection). TtRabD9 labels a vesicular structure at the base of the oral apparatus (cytostome) in addition to medium-sized heterogeneous puncta. L. TtRabD11 labels cytoplasmic puncta and, in some cells, small tubulovesicular structures near the cortex. M. TtRabD21 labels 1–3 vacuoles in the posterior that do not contain india ink. These may represent structures that are more abundant in starved cells, where this Rab is dramatically induced. O. (Projection of a z stack) TtRabD35 labels an anterior cortical cap, and also labels putative recycling endosomes (Figure S6I).
(2.55 MB TIF)

Figure S9 Phylogenetic relationships between Rabs in *T. thermophila* and in *H. sapiens*, *D. melanogaster*, *A. thaliana*, and *P. tetraurelia*. Phylogenetic analyses using three different approaches (maximum likelihood, Bayesian, and neighbor-joining) are shown. Bootstrap values below 50% are not shown. The trees include all predicted Rabs in *T. thermophila*, and *H. sapiens*, and selected Rabs from *D. melanogaster*, *A. thaliana*, and *P. tetraurelia*. Human (red arrows) and *Tetrahymena* Rabs (green arrows) associated with phagosomes are marked on the maximum likelihood tree.
(6.11 MB DOC)

Table S1 Fifty-four *Tetrahymena* Rabs are co-expressed in vegetative cells. Shown are signal intensities in arbitrary units (AU) for each Rab, as measured using whole genome microarrays for mRNAs isolated from cultures at successive time points under three different conditions: 1. growth medium [Ll (exponential growth) = ∼1×10^5^ cells/ml); Lm (deceleratory) = ∼3.5×10^5^ cells/ml); Lh (stationary) = ∼1×10^6^ cells/ml))]; 2. starvation (S0 to S24, corresponding to successive time points in hours) and; 3. conjugation (C0 to C18, corresponding to successive time points in hours). For each Rab, the stage showing highest expression is boxed. The expression data were obtained by [27] and are publicly accessible at http://tged.ihb.ac.cn/. Conserved Rabs are emboldened.
(0.16 MB XLS)

Table S2 Summary of all TtRabs based on the primary localization pattern, and also noting 2° (i.e., weaker) localization patterns.
(0.02 MB XLS)

Table S3 Forward and reverse primers used to amplify each of the Rab genes.
(0.03 MB XLS)

Video S1 TtRabD17-GFP brightly labels the oral apparatus near the cell anterior and, at the cell posterior, both phagosomes (one bright, one more dim) and the adjacent cytoproct on the plasma membrane. The movie play rate is 5× the collection rate, which was 1 frame/sec.
(2.06 MB MOV)

Video S2 TtRabD20-GFP, captured in three movies filmed in succession. RabD20-GFP labels late-stage phagosomes. The first movie captures the successive fusion of two phagosomes with the cytoproct, The second movie shows fusion of the third phagosome and the beginning of what appears to be membrane recovery, with gradual recruitment or retrieval of RabD20-GFP to newly forming vesicles at the same site. This process continues in the third movie. The phagosome membranes appear highly dynamic in these movies. The movie play rate is 10× the collection rate, which was 1 frame/sec.
(4.53 MB MOV)

Video S3 TtRabD20-GFP, captured in three movies filmed in succession. RabD20-GFP labels late-stage phagosomes. The first movie captures the successive fusion of two phagosomes with the cytoproct, The second movie shows fusion of the third phagosome and the beginning of what appears to be membrane recovery, with gradual recruitment or retrieval of RabD20-GFP to newly forming vesicles at the same site. This process continues in the third movie. The phagosome membranes appear highly dynamic in these movies. The movie play rate is 10× the collection rate, which was 1 frame/sec.
(8.96 MB MOV)

Video S4 TtRabD20-GFP, captured in three movies filmed in succession. RabD20-GFP labels late-stage phagosomes. The first movie captures the successive fusion of two phagosomes with the cytoproct, The second movie shows fusion of the third phagosome and the beginning of what appears to be membrane recovery, with gradual recruitment or retrieval of RabD20-GFP to newly forming vesicles at the same site. This process continues in the third movie. The phagosome membranes appear highly dynamic in these movies. The movie play rate is 10× the collection rate, which was 1 frame/sec.
(3.53 MB MOV)

Video S5 TtRabD3-GFP brightly labels phagosomes in the vicinity of the cytoproct (large bright bodies in cell posterior; see Figure 2F) and long tubules that extend from these phagosomes in the anterior direction, reaching the anterior of the cell. The labeling along these tubules is not continuous and appears to represent elongated vesicles moving along cytoplasmic microtubules. The movie play rate is 4× the collection rate, which was 2 frame/sec.
(5.97 MB MOV)

Video S6 Time-lapse movie of TtRab11B, showing abundant cytoplasmic vesicles and, at the cell anterior, a brightly labeled oral apparatus. Labeled vesicles can be seen moving along fibers that extend into the cytoplasm from the base of the oral apparatus. The vesicles move predominantly toward the oral apparatus. The movie play rate is 4× the collection rate, which was 2 frames/sec.
(4.86 MB MOV)

Video S7 TtRab6B-GFP, in the retrograde-Golgi clade, primarily localizes to puncta that are irregularly spaced along 1° meridians, and are concentrated in the cell anterior. These puncta are largely immobile, but a set of highly mobile, smaller vesicles or tubules are also seen, moving rapidly along what are likely to be cortical microtubules. The movie play rate is 4× the collection rate, which was 2 frame/sec.
(8.85 MB MOV)
