# Supplementary figures and images for: Comprehensive Analysis Reveals Dynamic and Evolutionary Plasticity of Rab GTPases and Membrane Traffic in Tetrahymena thermophila
Source: PLoS Genet. 2010 Oct 14;6(10):e1001155. doi: 10.1371/journal.pgen.1001155 (PMC2954822; doi:10.1371/journal.pgen.1001155)

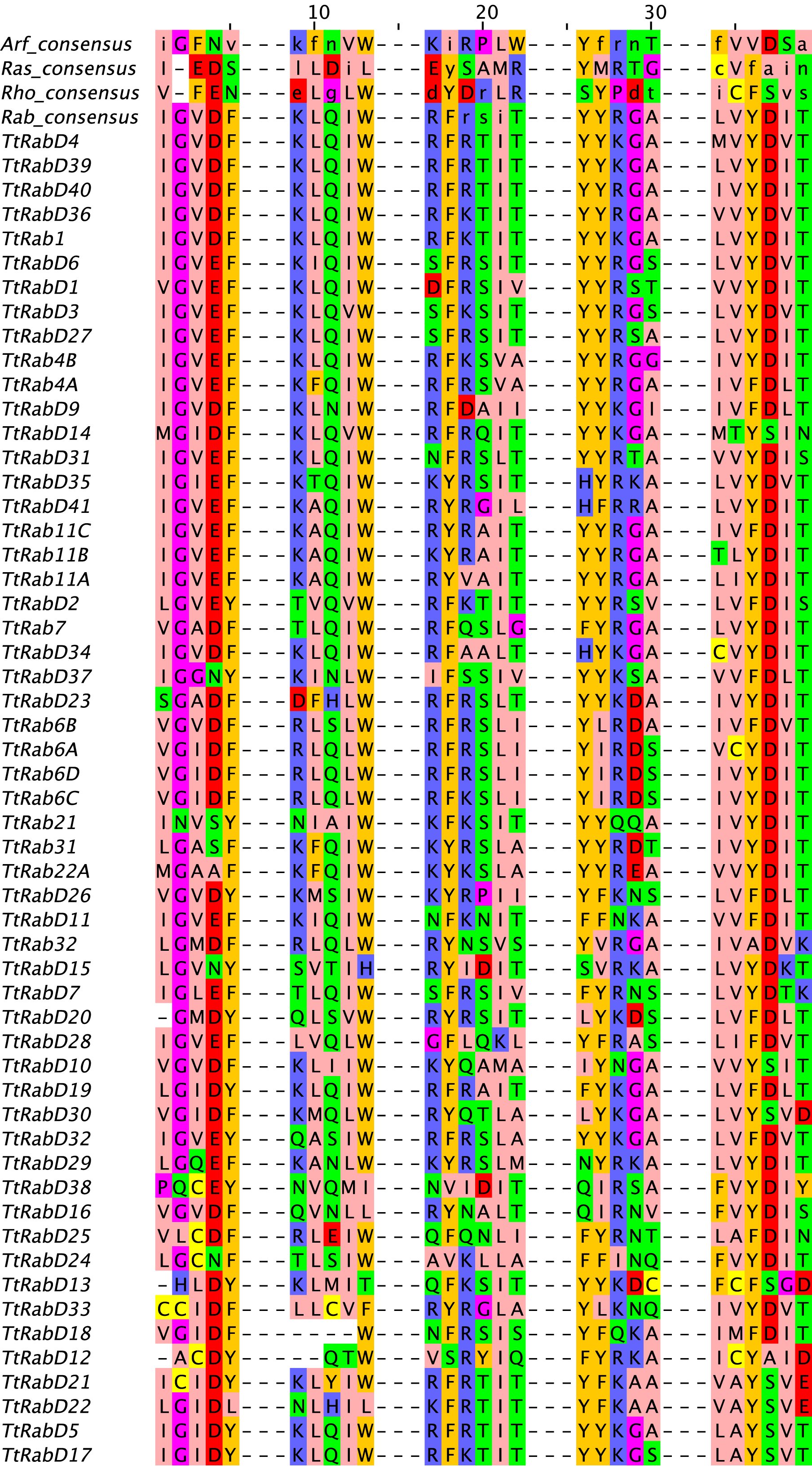

Supplement: Figure S1 — Multiple sequence alignment of all T. thermophila Rabs showing the five Rab-defining motifs. The consensus motifs for other classes of Ras-related GTPases are shown. The gaps shown between motifs do not represent the real spacing in the polypeptide sequences. (1.28 MB TIF) [file pgen.1001155.s001.tif]

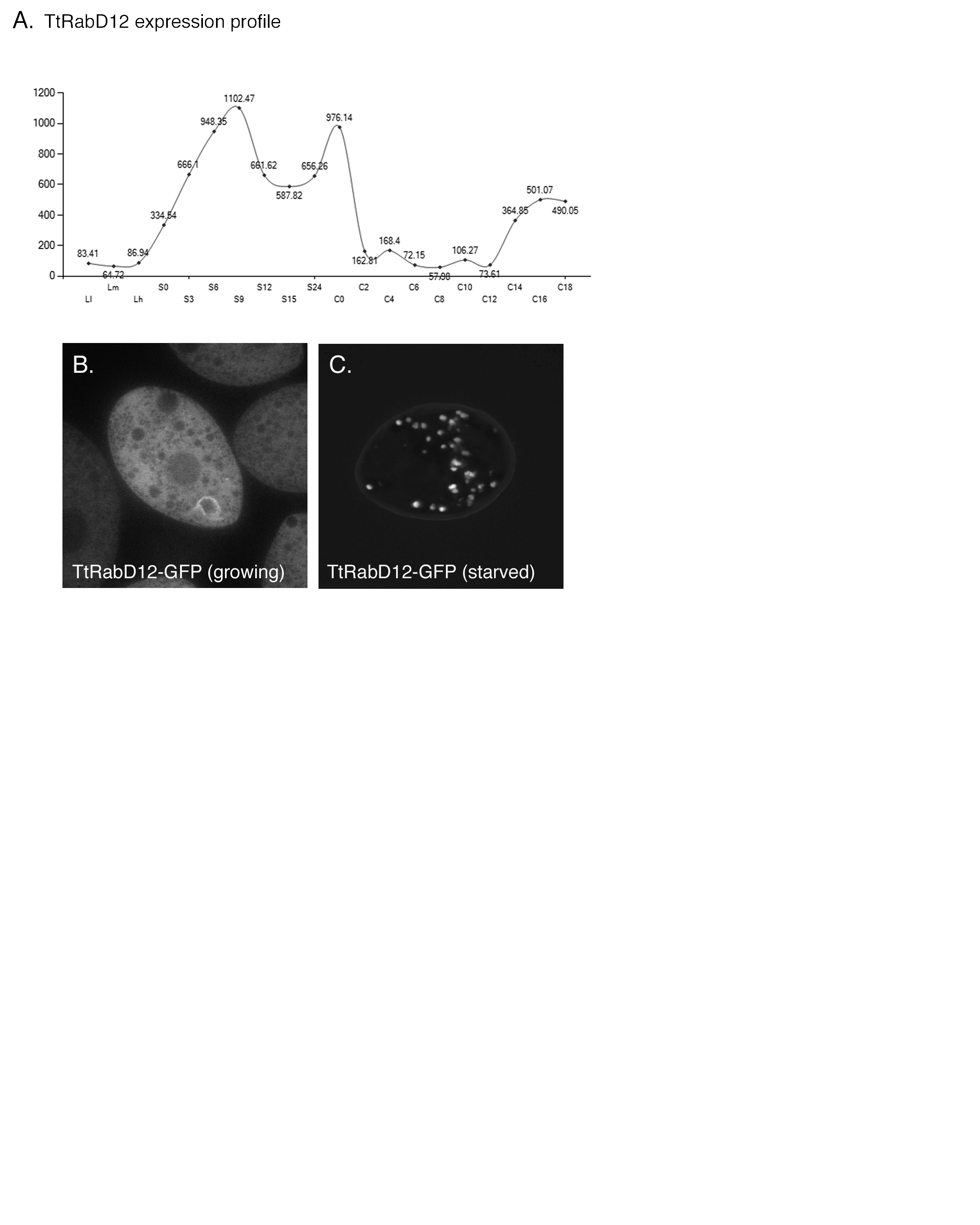

Supplement: Figure S2 — Example of stage-specific or -enhanced expression. Expression profile is from the Tetrahymena Gene Expression Database at http://tged.ihb.ac.cn/. Cell images are of live cells taken with a spinning disk confocal microscope, in S media (B) and DMC starvation media (C). A. TtRabD12 is predicted from expression levels to be stage-specific for starvation. B. TtRabD12-GFP shows indeterminate localization in growing cells. C. In starved cells, the Rab is tightly localized in cytosplasmic puncta. (0.45 MB TIF) [file pgen.1001155.s002.tif]

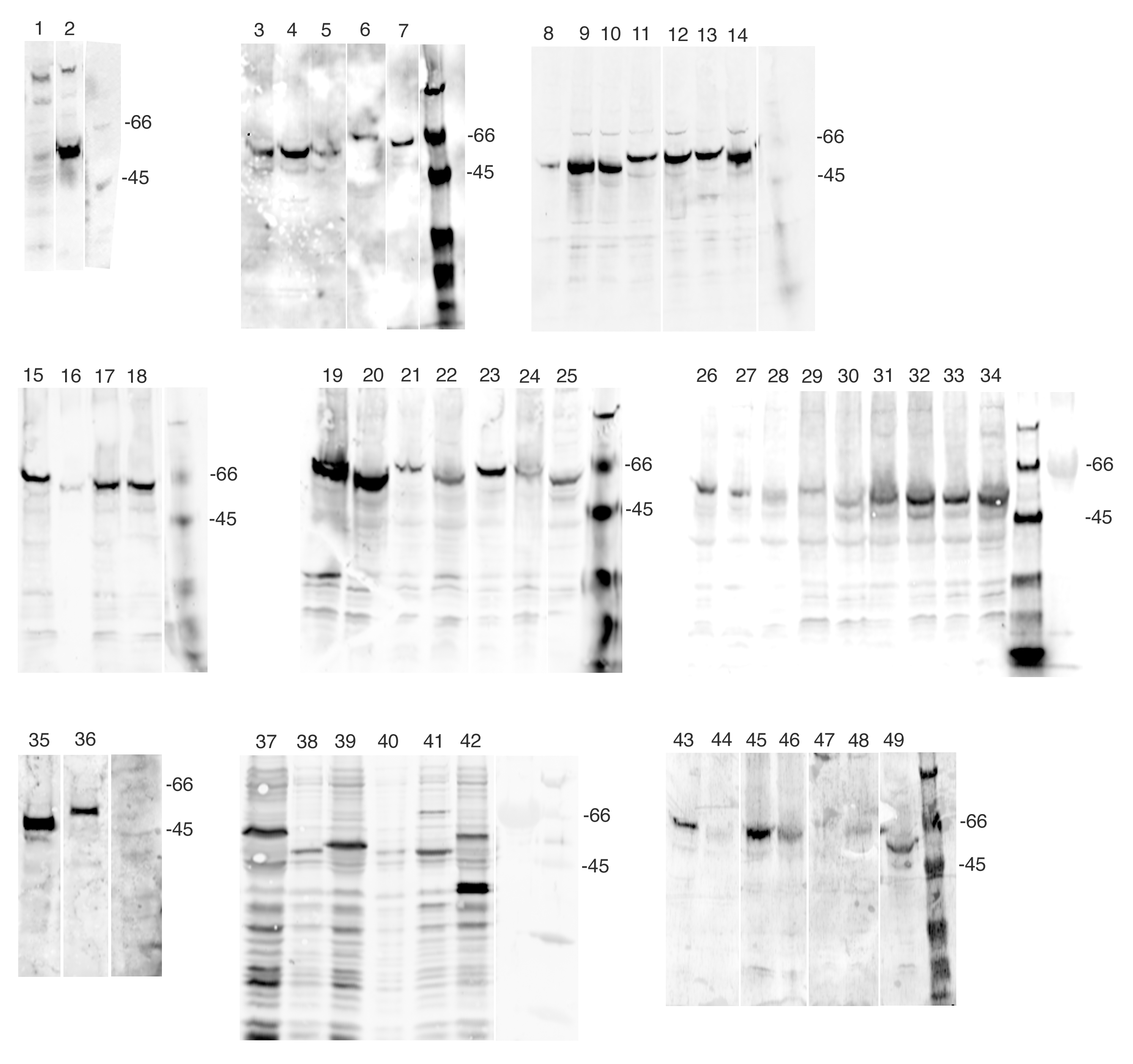

Supplement: Figure S3 — Expression of GFP-tagged proteins at the predicted sizes was confirmed by Western blotting of protein lysates, prepared as described in Methods. Blots were probed with a polyclonal anti-GFP antibody. SDS-PAGE low range molecular weight standards (Biorad) are labeled at 66 and 45 kD. Lane 1. TtRabD15; 2. D25; 3. D27; 4. Rab11B; 5. D3; 6. Rab6B; 7. D19; 8. D17; 9. Rab22A; 10. D11; 11. D35; 12. D34; 13. Rab21; 14. D38; 15. D39; 16. Rab6C; 17. Rab6D; 18. D6; 19. Rab4B; 20. D28; 21. D7; 22. D4; 23. D29; 24. D26; 25. D36; 26. Rab6A; 27. Rab4A; 28. D24; 29. Rab7; 30. D16; 31. D14; 32. D30; 33. D5; 34. D20; 35. D21; 36. D40; 37. D18; 38. Rab1; 39. Rab11A; 40. D41; 41. D12; 42. D31; 43. D32; 44. D9; 45. Rab32; 46. D13; 47. D10; 48. D2; 49. D33. Seven Rabs are not shown: RabD37 could not be cloned, Rab11C was not expressed at the predicted size; RabsD22 and D1 were not stable at levels high enough for either visualization or Western blotting; RabsD23 and 31, which when expressed at levels that showed distinct though dim localization signals, could not be detected by Western blot. (1.97 MB TIF) [file pgen.1001155.s003.tif]

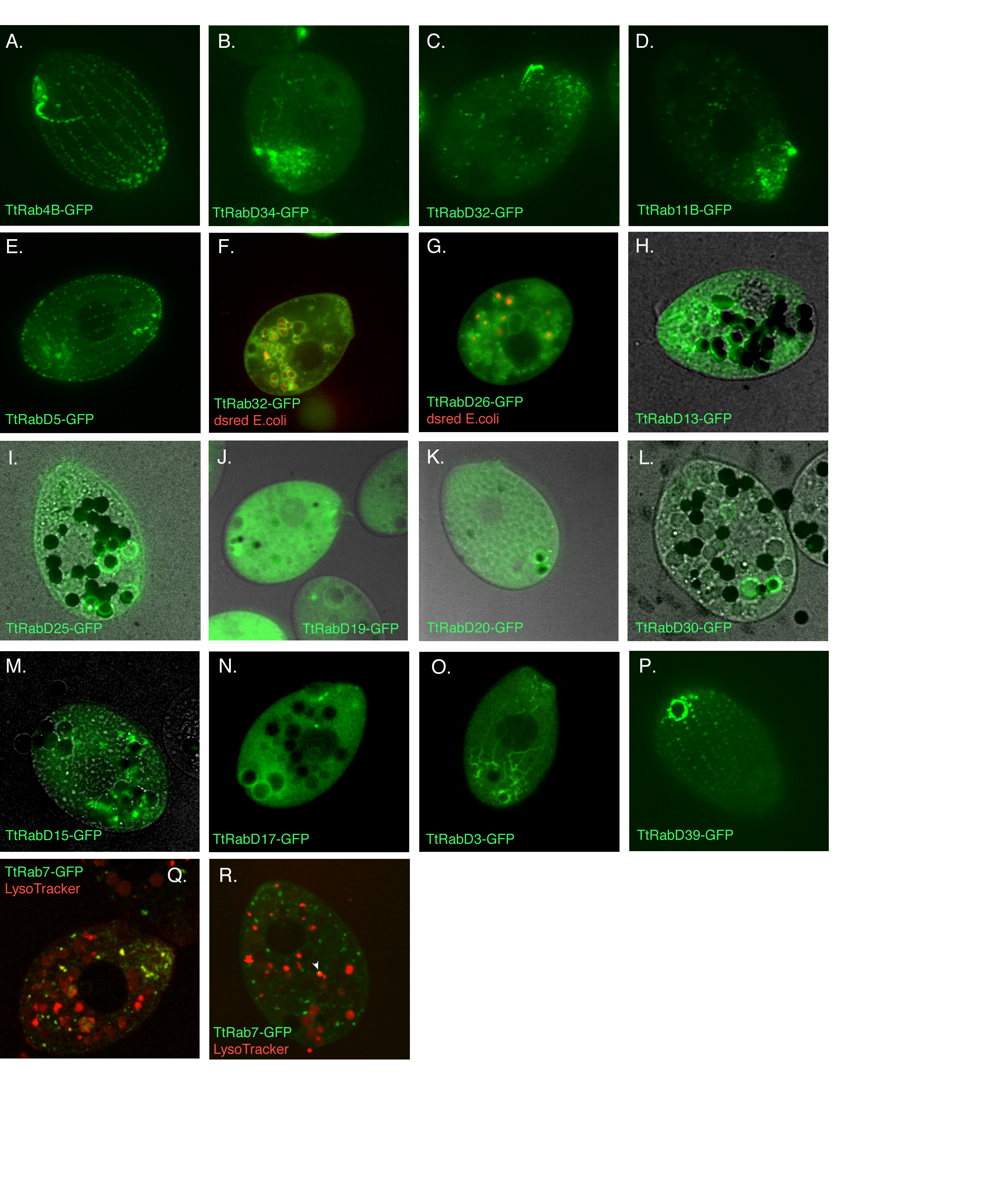

Supplement: Figure S4 — The set of Rabs associated with phagocytic uptake or digestion. All panels are confocal images of live cells following induction of GFP-Rab expression for 2 hours in S media, unless otherwise indicated. Green: TtRab-GFP. A–E. Rabs that label the oral apparatus. Additional observations: A. (a projection of a z-stack) TtRab4B labels the oral apparatus, primary meridians at the cortex, and bright vesicles in the posterior cytoplasm. D. TtRab11B labels part of the oral apparatus, including the deep fiber, and small vesicles concentrated in the anterior cytoplasm (movie, Video S6). E. (Projection of maximum intensities of a z stack). TtRabD5 puncta are strikingly mobile, especially near the oral apparatus. This Rab also shows localization to parasomal sacs, and at the contractile vacuole. F–O. Rabs that primarily label some or all phagosomes, visualized by uptake of dsRed-expressing bacteria (red) or india ink (black, shown as overlays with DIC channel. Additional observations: J. TtRabD19 secondarily labels 1° meridians including part of the oral apparatus. L. TtRabD30 labels irregularly spaced puncta at primary cortical meridians, with little or no overlap with a basal body marker. N. In addition to labeling the oral apparatus, TtRabD17 labels phagosomes in the cell posterior, and also small mobile vesicles. O. TtRabD3 localizes to phagosomes in the cell posterior and also to vesicles transported along cytoplasmic microtubules (movie, Video S5) P. TtRabD39 strongly labels the cytoproct (the plasma membrane zone where mature phagosomes fuse to egest undigested contents) and secondarily labels the nascent oral apparatus as well as 1° and 2° meridians. Q,R. For cells expressing TtRab7 in SPP medium (Q), LysoTracker (red) labels both phagosomes (larger red vacuoles) and smaller vesicles (red and yellow) that are likely to be lysosomes. TtRab7 colocalizes with the latter, which are concentrated in the anterior cytoplasm. R. In cells cultured in S medium, TtRab7 co-localizes [file pgen.1001155.s004.tif]

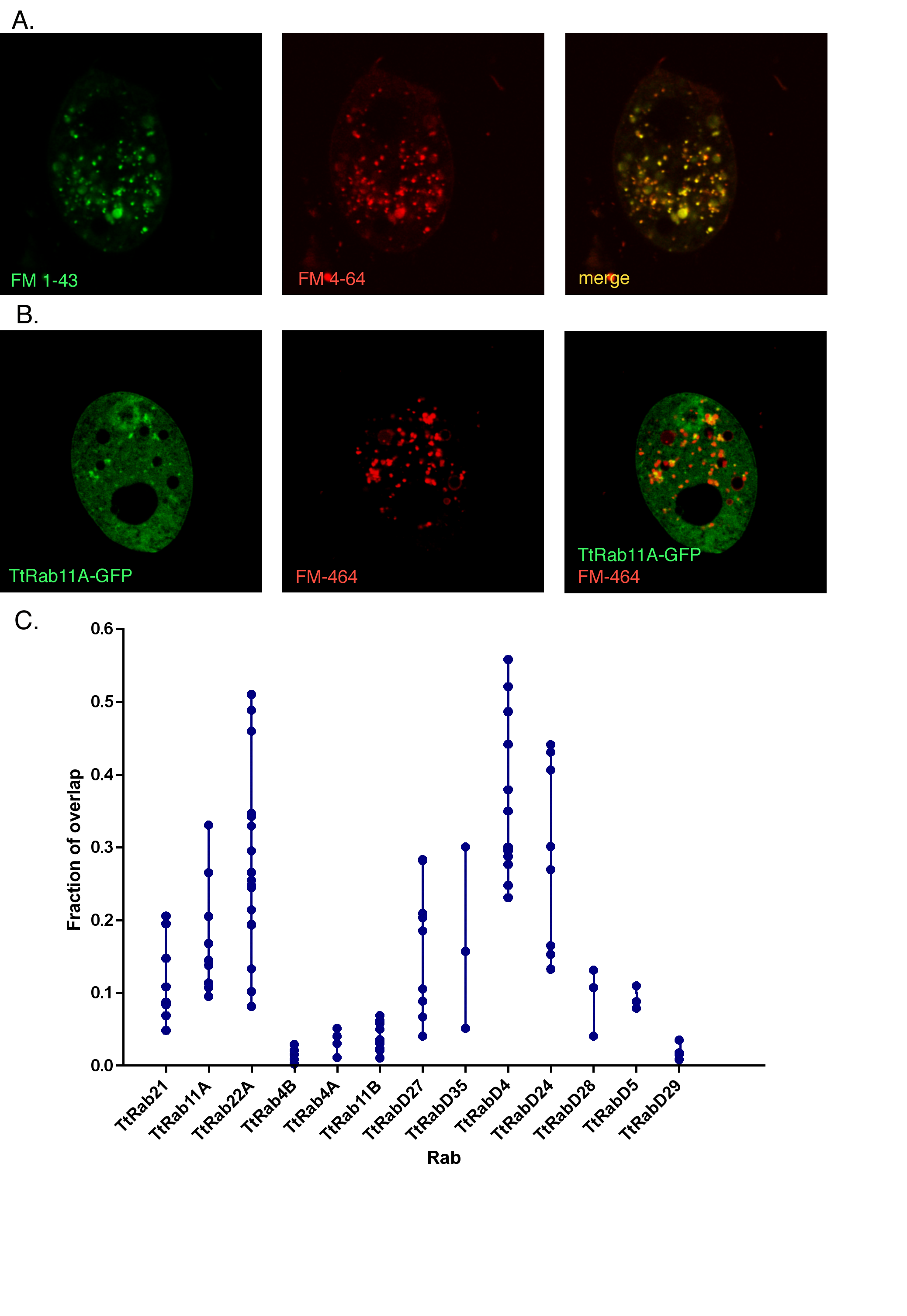

Supplement: Figure S5 — Colocalization of endocytic Rabs with lipophilic styryl dyes. A. Cells incubated for 60 min with FM 1-43 (green; shown previously to be an accurate endocytic tracer in Tetrahymena [42] and FM 4-64 (red) show near-complete overlap between the two dyes. B. The individual green and red channels, and the merge, shown for TtRab11A. C. Colocalization of FM 4-64 and GFP in cells expressing endocytic Rab-GFPs, quantified as described in Methods. For each line, each data point represents colocalization measured in a single cell. Complete overlap would register as 1.0 (on the ordinate). (0.93 MB TIF) [file pgen.1001155.s005.tif]

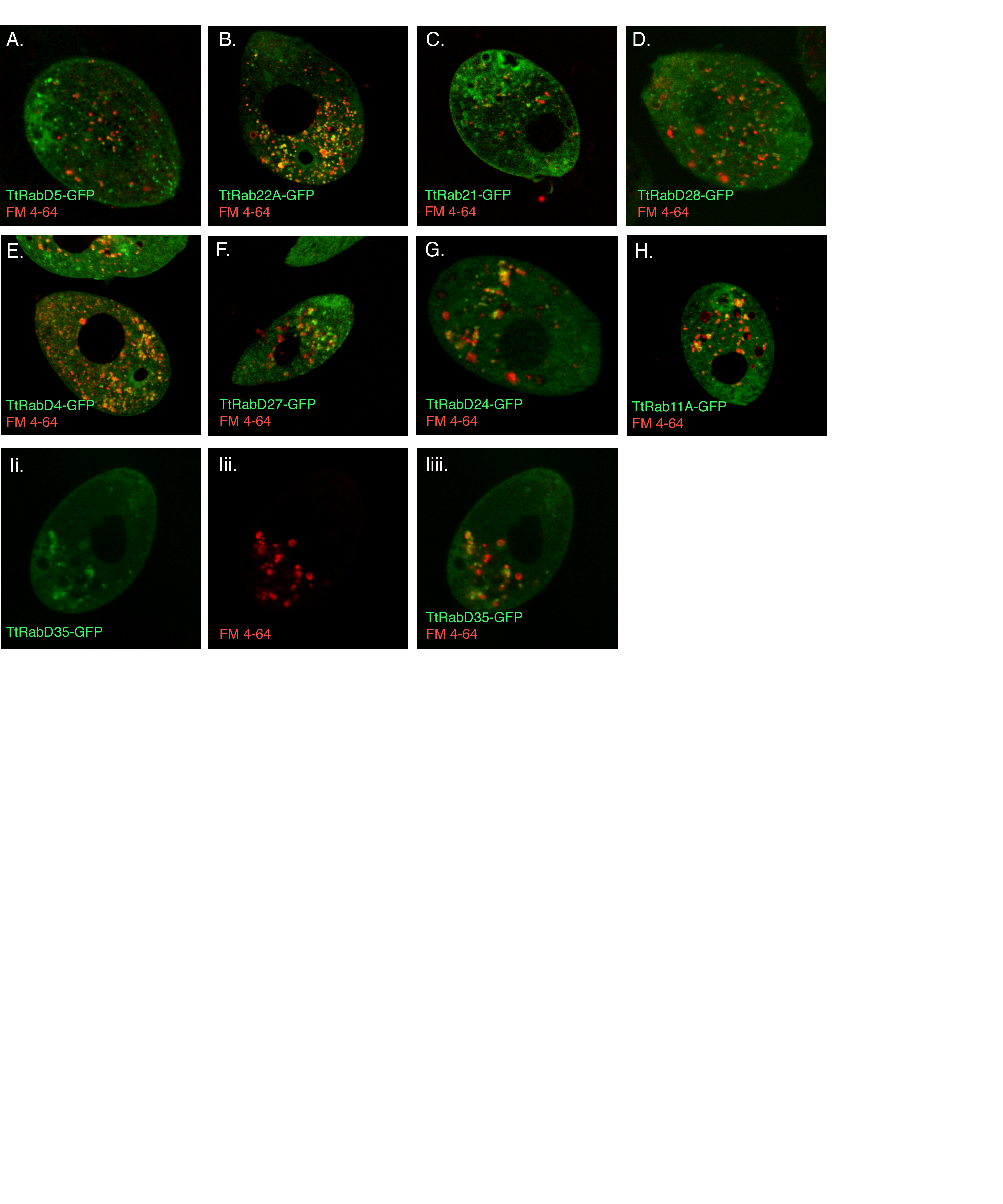

Supplement: Figure S6 — The set of Rabs associated with endocytosis. All panels are confocal images of live cells following induction of GFP-Rab expression for 2 hours in S media, unless otherwise indicated. Rab-GFP expression is in the green channel; FM4-64 is in the red channel. Images are single confocal slices. Additional observations: A. TtRabD5 also labels the base of the oral apparatus (see Figure S4). C. TtRab21 co-localizes with small FM 4-64-positive vesicles, including at the cortex, but not with larger FM 4-64-positive cytoplasmic vesicles. It also shows diffuse localization near the cortex and the contractile vacuole. D. TtRabD28 also labels the contractile vacuole. E. TtRabD4 labels some cortical puncta. G. TtRabD24 is exclusively cytoplasmic with no cortical signal. H. TtRab11A labels both vesicular and tubular endosomes. This Rab also appears concentrated in a zone around the contractile vacuole, and to a lesser extent at the plasma membrane and in the anterior cytoplasm. Panels Ii–Iiii represent the red, green and merged channels respectively for a TtRabD35-GFP expressing cell. This Rab also labels the cortex at the anterior end of the cell (see Figure S8). (3.08 MB TIF) [file pgen.1001155.s006.tif]

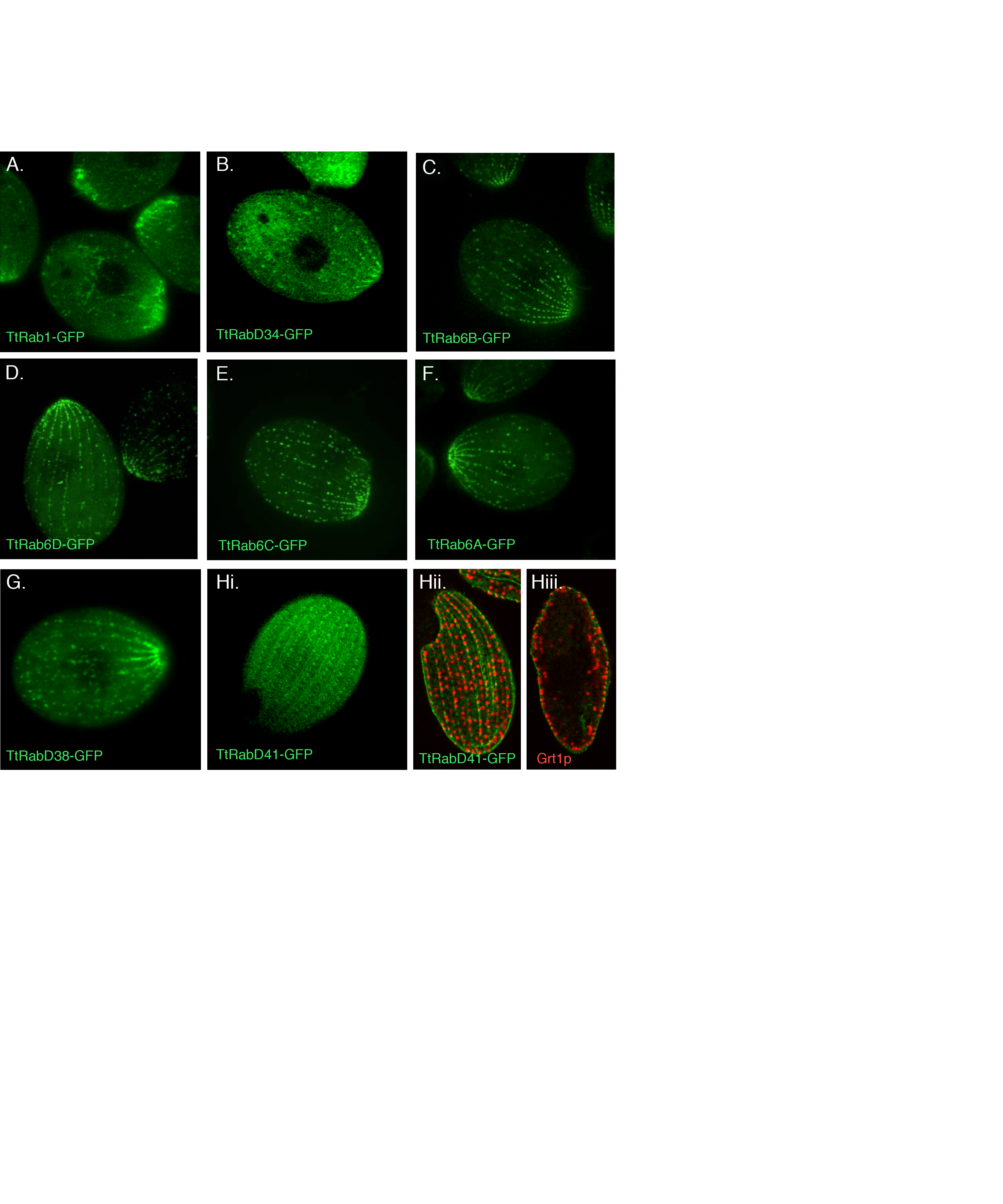

Supplement: Figure S7 — The set of Rabs associated with protein secretion. All panels are confocal images of live cells following induction of GFP-Rab expression for 2 hours in S media, unless otherwise indicated. A–B. TtRabs putatively associated with the ER or ER-to-Golgi traffic. C–G. TtRabs putatively associated with the Golgi. C,E,F are projections of z stack maximum intensities, while D is a single slice taken at the labeled meridians. All five Rabs label mobile puncta that are near but not at the cortex, which are brighter and more concentrated at the anterior end, as well as mobile puncta within in the cytoplasm (Video S7). Hi–Hiii. TtRabD41 shows extensive colocalization with docked dense core granules, for which Grt1p is a marker. (2.49 MB TIF) [file pgen.1001155.s007.tif]

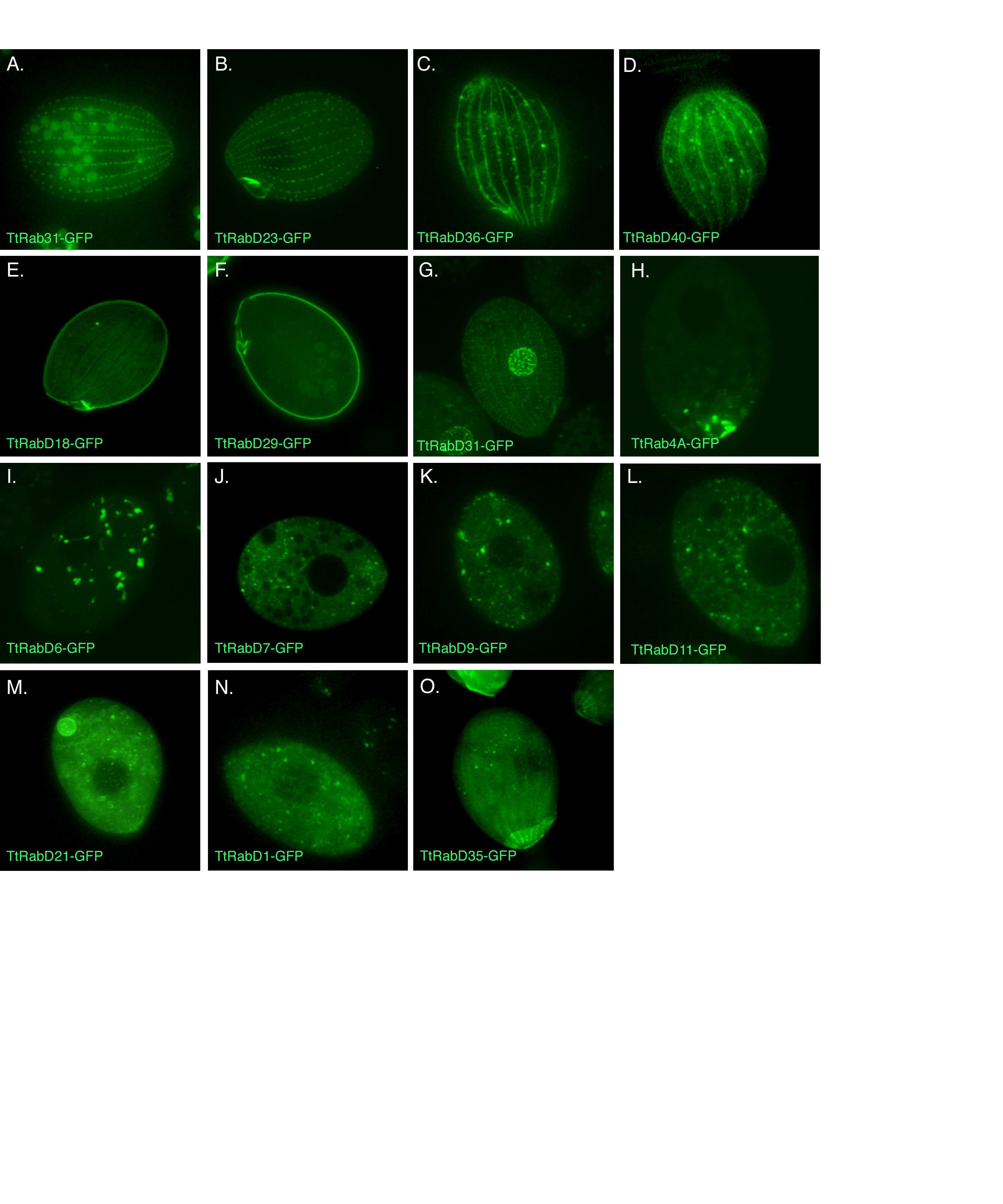

Supplement: Figure S8 — Fourteen TtRabs localize to the cell cortex, the macronuclear envelope, and other cell structures. All panels are confocal images of live cells following induction of GFP-Rab expression for 2 hours in S media, unless otherwise indicated. A. TtRab31 localization was limited to faintly fluorescent puncta at primary meridians. Because of the long exposure times needed to capture this signal, phagosomes in these cells are illuminated by autofluorescence. Shown is a projection of a z stack. B. TtRabD23 has a localization pattern that is very similar to basal bodies. C. TtRabD36, in addition to showing continuous labeling of 1° meridians, labels mobile cytoplasmic puncta. D. TtRabD40 appears to continuously label 1° meridians, and also labels bright puncta both at meridians and in the cytoplasm. E. TtRabD18 labels the cortex uniformly, but in many cells also shows a small number of bright cortical puncta and dim fluorescent cytoplasmic puncta. F. TtRabD29 shows uniform labeling of the cortex. G. TtRabD31 labels the macronuclear envelope brightly, and shows faint puncta along 1° meridians. H. TtRab4A brightly labels medium-sized puncta toward the posterior end of 1° meridians, which are strikingly concentrated near the cytoproct. It also labels the oral apparatus and small puncta along 1° meridians. Only the puncta near the cytoproct are mobile. I. TtRabD6 labels heterogeneous mobile cytoplasmic vesicles that show little or no overlap with FM4-64, and that move rapidly and directionally; some cells show labeled mobile tubules that extend along meridians or from the cortex into the cytoplasm. J. TtRabD7 labels heterogeneous vesicles that do not accumulate FM 4-64 and are concentrated in the cell posterior, with some labeled vesicles appearing to move along microtubule tracks. K. (Whole cell z projection). TtRabD9 labels a vesicular structure at the base of the oral apparatus (cytostome) in addition to medium-sized heterogeneous puncta. L. TtRabD11 labels cytoplasmic puncta [file pgen.1001155.s008.tif]

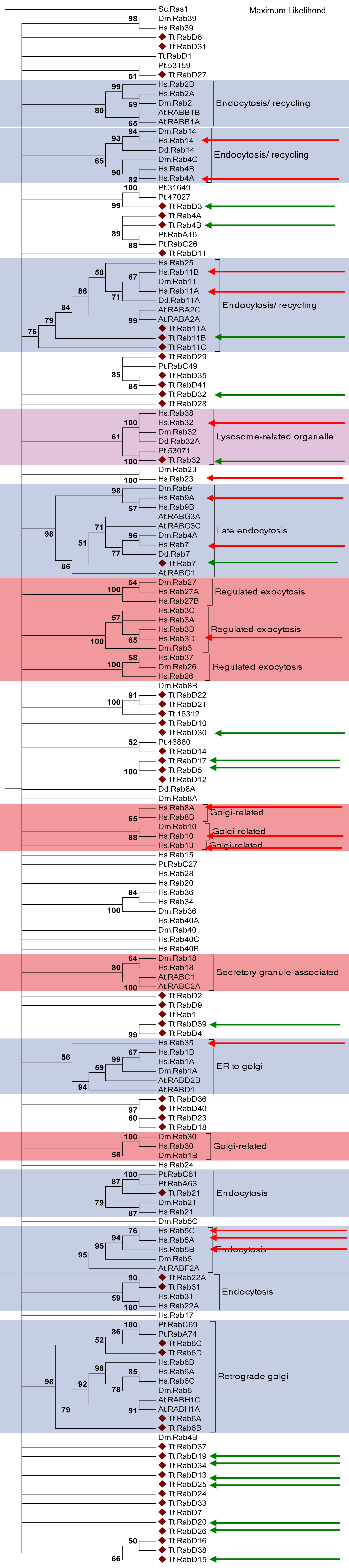


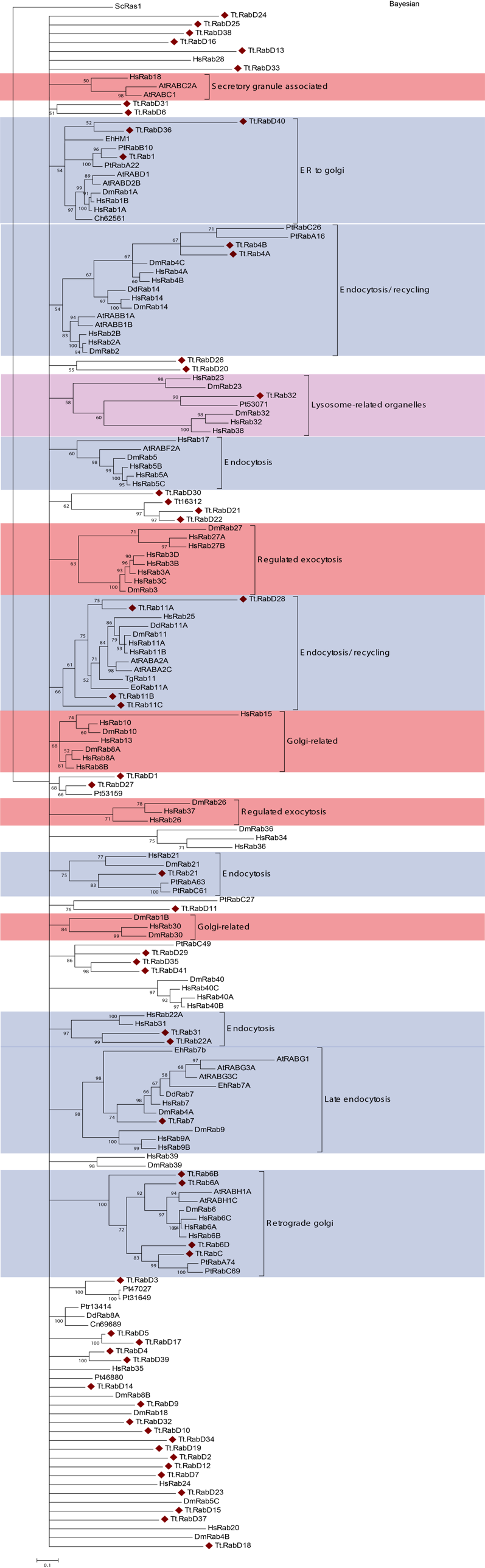


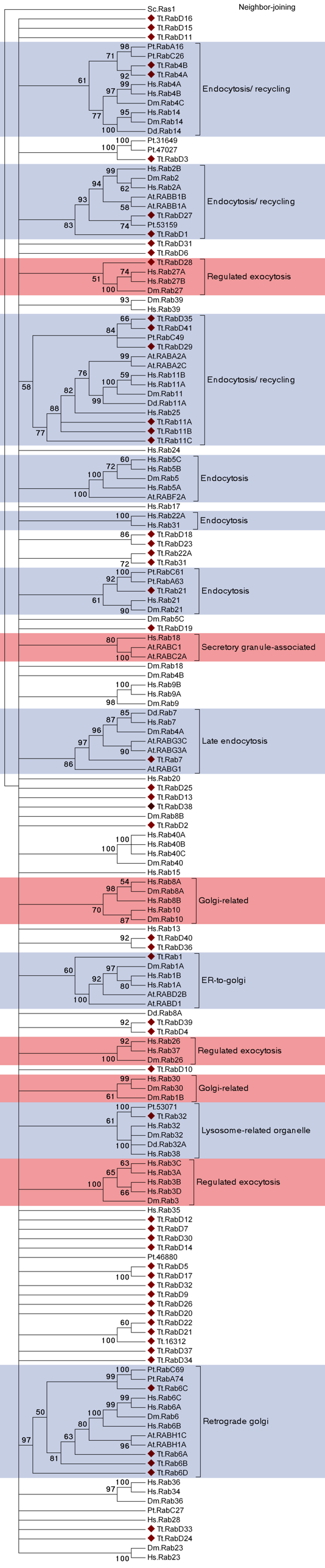

Supplement: Figure S9 — Phylogenetic relationships between Rabs in T. thermophila and in H. sapiens, D. melanogaster, A. thaliana, and P. tetraurelia. Phylogenetic analyses using three different approaches (maximum likelihood, Bayesian, and neighbor-joining) are shown. Bootstrap values below 50% are not shown. The trees include all predicted Rabs in T. thermophila, and H. sapiens, and selected Rabs from D. melanogaster, A. thaliana, and P. tetraurelia. Human (red arrows) and Tetrahymena Rabs (green arrows) associated with phagosomes are marked on the maximum likelihood tree. (6.11 MB DOC) [file pgen.1001155.s009.doc]
